# Isoetin from Isoetaceae Exhibits Superior Pentatransferase Inhibition in Breast Cancer: Comparative Computational Profiling with FDA-Approved Tucatinib

**DOI:** 10.3390/ph18050662

**Published:** 2025-04-30

**Authors:** Abdulaziz H. Al Khzem, Mansour S. Alturki, Ohood K. Almuzaini, Saad M. Wali, Mohammed Almaghrabi, Mohammed F. Aldawsari, Maram H. Abduljabbar, Reem M. Alnemari, Atiah H. Almalki, Thankhoe A. Rants’o

**Affiliations:** 1 Department of Pharmaceutical Chemistry, College of Pharmacy, Imam Abdulrahman Bin Faisal University, P.O. Box 1982, Dammam 31441, Saudi Arabia; msalturki@iau.edu.sa; 2 Department of Pharmacology and Toxicology, College of Pharmacy, Umm Al-Qura University, Makkah 24342, Saudi Arabia; okmuzaini@uqu.edu.sa (O.K.A.); smwali@uqu.edu.sa (S.M.W.); 3 Department of Pharmacognosy and Pharmaceutical Chemistry, Faculty of Pharmacy, Taibah University, Al Madinah Al Munawarah 30001, Saudi Arabia; mhmaghrabi@taibahu.edu.sa; 4 Department of Pharmaceutics, College of Pharmacy, Prince Sattam Bin Abdulaziz University, Al-Kharj 11942, Saudi Arabia; 5 Department of Pharmacology and Toxicology, College of Pharmacy, Taif University, Taif 21944, Saudi Arabia; maram.a@tu.edu.sa; 6 Department of Pharmaceutics and Pharmaceutical Technology, College of Pharmacy, Taif University, P.O. Box 11099, Taif 21944, Saudi Arabia; r.alnemari@tu.edu.sa; 7 Department of Pharmaceutical Chemistry, College of Pharmacy, Taif University, P.O. Box 11099, Taif 21944, Saudi Arabia; ahalmalki@tu.edu.sa; 8 Addiction and Neuroscience Research Unit, College of Pharmacy, Taif University, Al-Hawiah, Taif 21944, Saudi Arabia; 9 Department of Pharmacology and Toxicology, College of Pharmacy, University of Utah, Salt Lake City, UT 84112, USA; thankhoe.rantso@pharm.utah.edu; 10 Huntsman Cancer Institute, University of Utah, Salt Lake City, UT 84112, USA

**Keywords:** breast cancer resistance, Isoetin, tucatinib, WaterMap, binding free energy

## Abstract

**Background:** Breast cancer, the most prevalent cancer among women globally, develops primarily in the breast’s ducts or lobules. Drug resistance is a significant challenge in treating advanced cases, contributing to over 685,000 breast cancer-related deaths annually, and identifying novel compounds that inhibit key proteins is crucial for developing effective therapies. **Methods:** In this study, five transferase proteins with PDB IDs were selected due to their involvement in breast cancer: 1A52, 3PP0, 4EJN, 4I23, and 7R9V. Multitargeted docking studies were conducted using three different docking strategies and Molecular Mechanics/Generalized Born Surface Area (MM/GBSA) to calculate the binding affinities against the ZINC Natural compound library. Isoetin (ZINC000006523948), found mainly in Isoetaceae, was identified, and the results were compared with the Food and Drug Administration (FDA)-approved drug Tucatinib. In addition, molecular interaction fingerprints and pharmacokinetic profiling were evaluated. We also performed 5 ns WaterMap simulations to identify hydration sites and interactions, followed by 100 ns molecular dynamics (MD) simulations and MM/GBSA to assess the stability of the Isoetin–protein complexes. **Results:** The docking results indicated that Isoetin demonstrated superior binding and docking scores ranging from −9.901 to −13.903 kcal/mol compared to Tucatinib, which showed values between −4.875 and −10.948 kcal/mol, suggesting Isoetin’s potential efficacy as a therapeutic agent for breast cancer. Interaction fingerprints revealed significant interactions between Isoetin and key residues, including 28LEU, 12MET, 9PHE, 7ASP, 6ASN, and 6THR. The pharmacokinetics and DFT analysis of Isoetin supported its potential as a viable drug candidate. Furthermore, the 5 ns WaterMap simulations identified various hydration sites, and the 100 ns MD simulations showed that the Isoetin–protein complexes exhibited minimal deviations and fluctuations, indicating better stability than Tucatinib, and MM/GBSA confirmed Isoetin’s superior binding stability. **Conclusions:** Isoetin, a natural compound identified through in silico screening, demonstrates significant promise as a potential therapeutic agent for breast cancer as it outperforms the FDA-approved drug Tucatinib, the respective native and FDA-approved drug. However, experimental validation is necessary before considering Isoetin for clinical use.

## 1. Introduction

Breast cancer is one of the most prevalent forms of cancer, affecting millions of women worldwide. It begins when abnormal cells in the breast tissue grow uncontrollably, often forming a lump or mass. While it primarily affects women, men can also develop breast cancer, though at a much lower rate [1,2]. The disease is not a single entity but a collection of subtypes with different molecular characteristics, making it highly complex and challenging to treat uniformly. The global burden of breast cancer is staggering, with the number of cases rising every year. In 2020 alone, there were approximately 2.3 million new cases diagnosed, and it accounted for nearly 685,000 deaths worldwide, and by 2050, breast cancer cases are expected to increase significantly due to ageing populations, lifestyle changes, and environmental factors, potentially leading to over three million new cases annually [3]. The rise in incidence is attributed to factors like hormonal imbalances, genetic predisposition, exposure to carcinogens, and lifestyle choices such as alcohol consumption, poor diet, and lack of physical activity. Early detection and treatment have improved survival rates, but the increasing number of cases means that a large number of patients will continue to succumb to the disease. Breast cancer starts at the cellular level, when mutations occur in key genes responsible for cell growth and division. These mutations can be inherited or acquired due to environmental influences [4]. The most commonly mutated genes in breast cancer include BReast CAncer gene 1 (*BRCA1)* and BReast CAncer gene 2(*BRCA2)*, tumour-suppressor genes that typically help repair DNA damage. When these genes are defective, cells accumulate genetic errors and eventually become cancerous. Initially, the cancer may be localised to the breast tissue, but as it progresses, it can invade nearby lymph nodes and spread to distant organs such as the liver, lungs, bones, and brain [5]. The prognosis of breast cancer depends on various factors, including the stage at diagnosis, molecular subtype, and response to treatment. Patients diagnosed at an early stage, when the tumour is still localised, have a much higher survival rate than those diagnosed with metastatic disease. Common diagnostic methods include mammography, ultrasound, Magnetic Resonance Imaging (MRI) and biopsy, which help determine the presence and characteristics of the tumour [6]. In recent years, molecular profiling techniques, such as next-generation sequencing and immunohistochemistry, have allowed for more precise breast cancer subtyping, enabling personalised treatment approaches [5,7].

One of the biggest challenges in breast cancer treatment is drug resistance, which significantly reduces the effectiveness of standard therapies. Resistance can be intrinsic, meaning the cancer cells are inherently unresponsive to a particular drug, or acquired, where the cells initially respond but later develop mechanisms to evade treatment [8,9]. Chemotherapy, hormone therapy, and targeted therapy have been instrumental in treating breast cancer, but many patients eventually develop resistance, leading to disease recurrence and progression [10]. The underlying mechanisms of drug resistance are diverse, including genetic mutations, activation of alternative signalling pathways, increased drug efflux, and modifications in the tumour microenvironment. This resistance limits the effectiveness of many standard treatments and leaves patients with fewer therapeutic options [11]. Minimising drug resistance requires a multi-pronged approach, including combination therapies, novel drug delivery systems, and developing drugs that simultaneously target multiple pathways [12]. One promising strategy is the design of multitargeted drug candidates that can inhibit several key proteins involved in breast cancer progression, reducing the likelihood of resistance development and improving treatment efficacy [13,14,15]. Several key proteins play a crucial role in breast cancer development and progression. Estrogen receptor alpha (ERα, PDB ID: 1A52) is a primary driver of hormone receptor-positive breast cancer [16]. It regulates gene expression and promotes the growth of breast cancer cells in response to estrogen. The kinase domain of human epidermal growth factor receptor 2 (HER2) (PDB ID: 3PP0) is another critical target, as HER2-positive breast cancer is known for its aggressive nature and high recurrence rate. HER2 amplification leads to uncontrolled cell proliferation and survival [17]. RAC-alpha serine/threonine-protein kinase 1 (AKT1) (PDB ID: 4EJN) is a central player in the PI3K/AKT signalling pathway, which promotes cell survival and growth. Hyperactivation of this pathway is commonly observed in breast cancer and contributes to resistance against many therapies [18]. The epidermal growth factor receptor (EGFR, PDB ID: 4I23) kinase domain is another important target, as EGFR is frequently overexpressed in triple-negative breast cancer, a highly aggressive and difficult-to-treat subtype [19]. Finally, Phosphatidylinositol-4,5-bisphosphate 3-kinase catalytic subunit alpha (PIK3CA) (PDB ID: 7R9V) is a catalytic subunit of PI3K and is frequently mutated in breast cancer, driving tumour progression through enhanced signalling activity [20]. A single multitargeted drug candidate capable of simultaneously inhibiting these key proteins could provide a revolutionary approach to breast cancer treatment. Instead of targeting one specific pathway, such a drug would disrupt multiple signalling cascades essential for tumour growth and survival. By simultaneously blocking estrogen receptor activity, HER2 signalling, AKT1-mediated survival pathways, EGFR activation, and PI3K-driven proliferation, the drug would prevent cancer cells from using alternative pathways to evade treatment. This approach could enhance therapeutic efficacy and reduce the likelihood of drug resistance [21,22]. Multitargeted drug design uses structural biology, computational modelling, and high-throughput screening to develop molecules that bind effectively to multiple targets. This strategy minimises the chances of cancer cells adapting and surviving, offering a more durable and comprehensive treatment option [9]. Such drugs can be designed using fragment-based drug discovery, molecular docking, and artificial intelligence-driven optimisation to ensure high specificity and potency [23].

Tucatinib is a highly selective, small-molecule tyrosine kinase inhibitor that targets the HER2 pathway, which plays a central role in the development and progression of breast cancer [24,25]. HER2 is overexpressed in approximately 15–20% of breast cancers, known as HER2-positive breast cancer, and is associated with aggressive tumour growth and poor prognosis. Tucatinib inhibits HER2 receptor signalling by blocking the receptor’s kinase activity, thereby preventing downstream signalling cascades that drive cancer cell proliferation, survival, and metastasis [26,27]. Unlike other HER2-targeted therapies, such as Trastuzumab and Lapatinib, Tucatinib has a unique advantage due to its high selectivity for HER2 over other EGFR family receptors, which reduces off-target effects and potentially limits side effects. Tucatinib works by selectively binding to the intracellular tyrosine kinase domain of HER2, blocking its autophosphorylation and interrupting its ability to activate the PI3K/Akt and MAPK signalling pathways. These pathways are critical for cell survival, division, and tumour progression. In clinical studies, Tucatinib has demonstrated significant efficacy in combination with Trastuzumab and Capecitabine for patients with advanced, HER2-positive breast cancer, particularly in those with brain metastases, where traditional treatments are less effective [24,28]. By effectively targeting HER2-driven signalling while minimising off-target effects, Tucatinib represents a promising therapeutic option for HER2-positive breast cancer, including those with refractory disease or central nervous system involvement [29]. Developing a single multitargeted drug candidate for breast cancer involves extensive research, but the potential benefits are enormous. It could streamline treatment regimens, reduce side effects associated with combination therapies, and provide patients with a more effective and lasting solution [30,31]. As breast cancer cases continue to rise globally, innovative approaches like multitargeted drug design will be essential in overcoming therapeutic resistance and improving patient outcomes. With advancements in structural biology, computational modelling, and precision medicine, the future of breast cancer treatment may be defined by highly selective, multitargeted therapies capable of addressing the complexities of this disease at a molecular level [23]. However, despite the promise of multitargeted drug design, several challenges must be addressed to make this approach successful [14,32,33]. One major issue is identifying compounds that can effectively bind to multiple targets without losing potency or selectivity. Designing a molecule that maintains a high affinity for different proteins while avoiding off-target effects requires a deep understanding of each target’s structural and chemical properties. Various computational methods have become integral to the drug discovery pipeline to overcome these challenges, including molecular docking, density functional theory (DFT), interaction fingerprints, WaterMap, molecular dynamics (MD)simulations, and binding free energy calculations with Molecular Mechanics/Generalized Born Surface Area (MM/GBSA) [34,35]. These computational methods work synergistically to accelerate the discovery of multitargeted drug candidates by narrowing the list of promising compounds before experimental validation.

**Figure 1:**
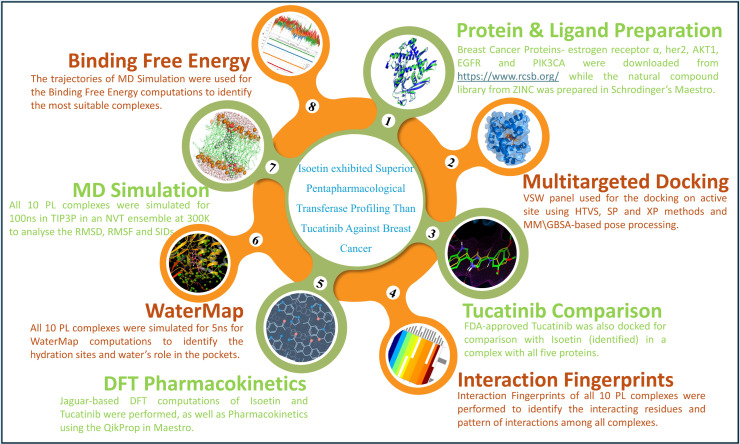
The workflow of the complete study shows the methods followed to identify Isoetin, validate its pentapharmacological transferase potency against multiple breast cancer proteins, and compare it with the FDA-approved drug Tucatinib. The link for Protein & Ligand Preparation method can be accessed here: https://www.rcsb.org/.

In this study, we performed multitargeted docking studies of the ZINC natural compound library against breast cancer proteins to identify the best candidate and compared the results with the Food and Drug Administration (FDA)-approved Tucatinib drug. Further, we extended our studies using interaction fingerprints, DFT, pharmacokinetics, WaterMap, and MD simulation, followed by the binding free energy computations for identifying the best poses. The whole workflow is shown in Figure 1.

## 2. Results

The multitargeted docking strategy has led to the identification of Isoetin as a natural drug candidate for breast cancer. We compared the results with Tucatinib, an FDA-approved drug used for breast cancer. The results for each step are provided below.

### 2.1. Validation and Analysis of Protein Structures

**Figure 2:**
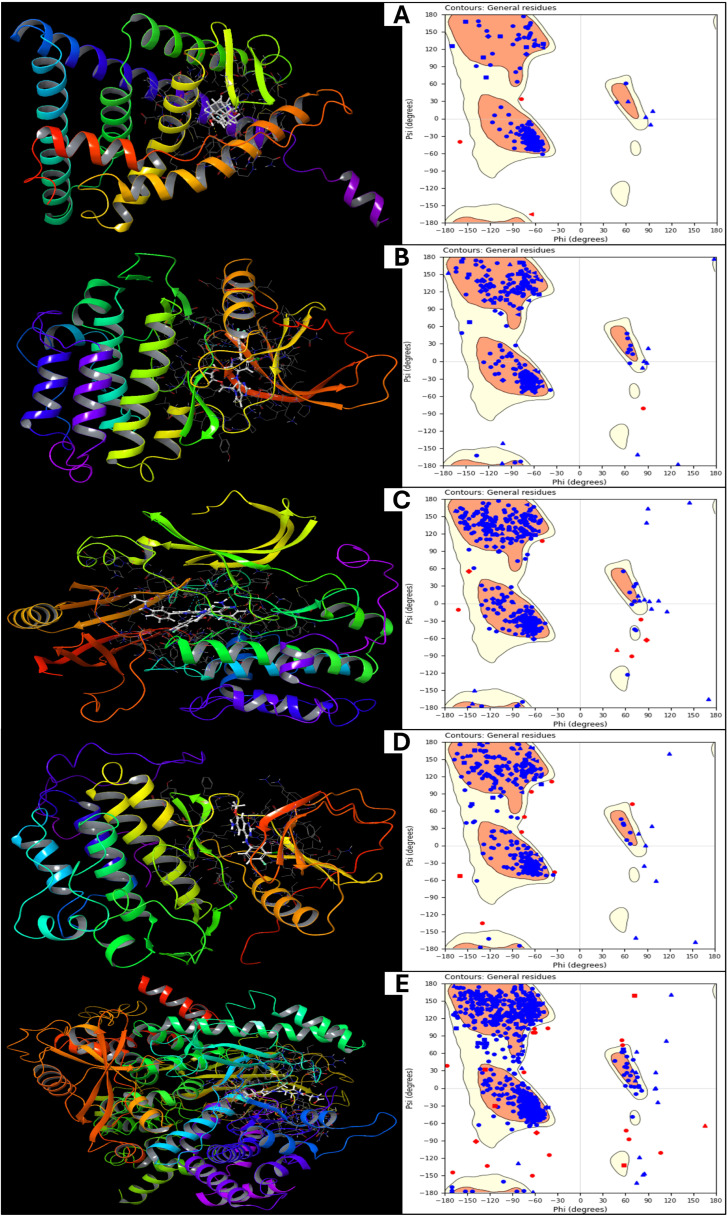
Showing the prepared 3D structures with native ligand bound positions and Ramachandran plot for (**A**) 1A52, (**B**) 3PP0, (**C**) 4EJN, (**D**) 4I23, and (**E**) 7R9V.

The final energy calculations for the prepared protein structures are analysed using Schrödinger’s Maestro 2024-2 providing detailed insights into their thermodynamic stability. The energy components for the five protein structures (PDB IDs: 1A52, 3PP0, 4EJN, 4I23, and 7R9V) are analysed in detail. The total energy of the proteins is identical to their potential energy, as the kinetic energy is zero due to the system being in an optimised, static state at absolute zero temperature (0 K). The values range from −1002.69 kcal/mol (1A52) to −3595.09 kcal/mol (7R9V), indicating different levels of structural stability, with 7R9V being the most stabilised protein among the five. We included bond stretch energy, angle bending energy, torsion angle energy, and restraining energy for the bonded energy components. The bond stretch energy varies from 113.35 kcal/mol (1A52) to 452.78 kcal/mol (7R9V), reflecting the energy required to maintain ideal bond lengths. A higher value suggests greater internal strain in the protein, while the angle bending energy values increase with protein complexity, ranging from 505.49 kcal/mol (1A52) to 2004.06 kcal/mol (7R9V), indicating significant angular distortions in larger proteins. The torsion angle energy represents rotational strain in the molecular framework, with 4EJN showing the highest torsional energy (907.78 kcal/mol), suggesting a highly flexible backbone, while 1A52 has the lowest at 351.93 kcal/mol, and the restraining energy in all proteins is 0 kcal/mol, confirming that no external positional constraints were applied. The non-bound interaction energies include 1,4 Lennard-Jones energy, 1,4 electrostatic energy, and hydrogen bond energy. The 1,4 Lennard-Jones energy is a short-range van der Waals interaction energy that ranges from 1141.75 kcal/mol (1A52) to 4386.65 kcal/mol (7R9V), indicating that larger proteins with more internal contacts exhibit higher values. 1,4 Electrostatic energy is an interaction energy that fluctuates between 343.99 kcal/mol (3PP0) and 1421.34 kcal/mol (7R9V), reflecting varying charge distributions within each protein. The Lennard-Jones energy has the overall van der Waals interaction energy, which is highly negative, ranging from −2298.12 kcal/mol (1A52) to −8944.51 kcal/mol (7R9V), signifying strong stabilising forces. The electrostatic energy is the strongest stabilising force that comes from electrostatic interactions, with values from −1291.57 kcal/mol (1A52) to −4720.78 kcal/mol (7R9V), reinforcing the importance of charged residues and polar interactions in structural stability. The H-bond energy is consistently 0 kcal/mol, implying that explicit hydrogen bonds were not separately accounted for in this calculation, possibly due to the chosen force field settings. The computed energy values provide critical insights into the stability, flexibility, and noncovalent interactions governing the prepared protein structures. Notably, 7R9V exhibits the lowest total energy (−3595.09 kcal/mol), indicating its highest stability, likely due to its extensive van der Waals and electrostatic interactions. In contrast, 1A52 has the least negative total energy (−1002.69 kcal/mol), suggesting lower stability in its optimised state. Bonded energy components, including bond stretch, angle bending, and torsion angle energy, reveal the internal strain within each protein. Further, Figure 2 shows the prepared structures with native ligands and a Ramachandran plot for the prepared proteins, to verify the structure suitability. The highest values in 7R9V suggest a complex, tightly packed structure with significant conformational strain, while 1A52 has minimal strain due to its more minor and potentially less constrained conformation. The Lennard-Jones and electrostatic energies are dominant contributors to protein stability, with strong, attractive interactions (negative values) balancing repulsive forces. Interestingly, the hydrogen bond energy was recorded as zero for all structures, possibly due to the implicit treatment of H-bonds within the force field. Further validation through explicit hydrogen bond analysis or molecular dynamics simulations would provide a more comprehensive understanding of protein stability. These findings underscore the role of van der Waals forces, electrostatics, and torsional flexibility in determining protein stability. A deeper analysis incorporating solvent effects, molecular dynamics, and binding free energy calculations would further refine these insights, making them more relevant for drug discovery and structural biology applications.

### 2.2. Analysis of Multitargeted Molecular Docking Results and Control Comparison

**Figure 3:**
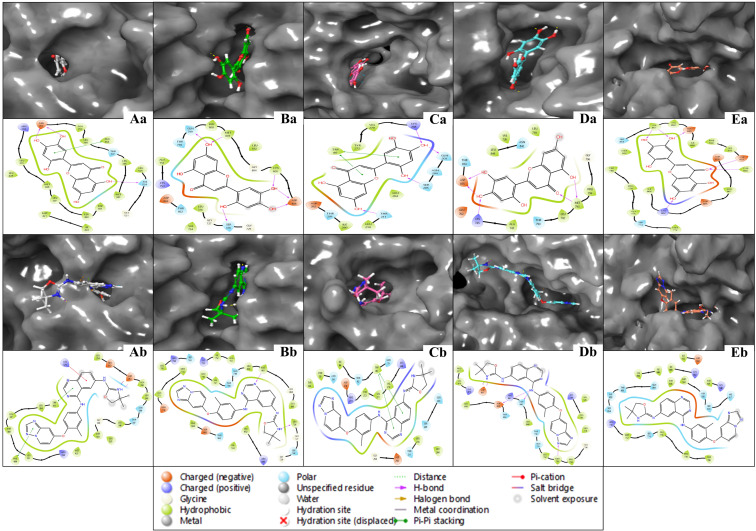
Showing the 3D docked poses and ligand interaction diagram (2D) of docked Isoetin (identified candidate) with proteins having PDB IDs (**Aa**) 1A52, (**Ba**) 3PP0, (**Ca**) 4EJN, (**Da**) 4I23, and (**Ea**) 7R9V, and Tucatinib in complex with (**Ab**) 1A52, (**Bb**) 3PP0, (**Cb**) 4EJN, (**Db**) 4I23, and (**Eb**) 7R9V for comparative understanding.

The multitargeted docking studies have identified Isoetin as a pentapharmacological transferase drug candidate that was compared with the standard drug Tucatinib against five protein targets: 1A52, 3PP0, 4EJN, 4I23, and 7R9V. The gridbox parameters were optimised for each protein to ensure accurate docking. The resolution of the proteins varied from 2.19 Å to 2.8 Å. The gridbox centre and range were adjusted for each structure to accommodate the binding pocket. The interaction profiles for each complex were analysed regarding hydrogen bonding, hydrophobic interactions, π–π stacking, salt bridges, and electrostatic interactions, revealing notable differences between how Isoetin binds compared to the FDA-approved Tucatinib. In the 1A52 complex, Isoetin forms hydrogen bonds with Glu796 and Lys753, contributing to strong electrostatic interactions, and engages several Leu and Met residues (e.g., Leu800, Met801) through hydrophobic interactions. In contrast, Tucatinib forms hydrogen bonds with Glu796 and interacts with Leu800, Met801, and Met793 via hydrophobic forces but additionally benefits from π-stacking interactions, which Isoetin lacks. Both compounds share key hydrogen bonding with Glu796, but Isoetin’s additional interaction with Lys753 might offer added stability. For the 3PP0 complex, Isoetin establishes hydrogen bonding with Asp262, Gly729, and Ser806 while interacting with Leu852, Leu800, and Cys805 through hydrophobic forces. Tucatinib shares hydrogen bonds with Asp262 and Ser806 but enhances its stability with π–π stacking interactions, which Isoetin does not exhibit. The key hydrogen bonds are similar, but Tucatinib’s use of π-stacking could stabilise its binding. In the 4EJN complex, both Isoetin and Tucatinib form hydrogen bonds with Asp855 and Gln792, but Isoetin engages with strong hydrophobic interactions involving Leu844, Leu264, and Thr211. At the same time, Tucatinib also utilises π–π stacking and halogen bonding to stabilise its binding further. These additional interactions in Tucatinib potentially make its binding stronger than that of Isoetin. For the 4I23 complex, Isoetin forms hydrogen bonds with Thr796 and interacts with hydrophobic Leu residues, while Tucatinib engages in similar hydrogen bonds but adds π–π stacking and pi-cation interactions that significantly enhance its binding strength. Isoetin lacks these additional interactions, particularly the π–π stacking, which could result in a weaker binding profile than Tucatinib. In the 7R9V complex, Isoetin forms hydrogen bonds with Glu796 and Thr798, with Leu-rich hydrophobic interactions contributing to binding stability. Tucatinib also forms hydrogen bonds, but it benefits from π–π stacking and solvent exposure compensation, which enhances its binding affinity. Isoetin’s electrostatic interaction with Glu796 is strong, but Tucatinib’s additional π-stacking interactions likely improve its overall stability. Further, Figure 3 has a 3D and 2D ligand interaction diagram for Isoetin and Tucatinib for a comparative analysis. For the identified ligand, Isoetin, the docking scores were as follows: −10.397 kcal/mol for 1A52, −10.399 kcal/mol for 3PP0, −9.901 kcal/mol for 4EJN, −9.639 kcal/mol for 4I23, and −13.903 kcal/mol for 7R9V. The MM/GBSA ΔG_binding free energy values showed a strong binding affinity, with −31.81 kcal/mol for 1A52, −37.04 kcal/mol for 3PP0, −47.31 kcal/mol for 4EJN, −36.8 kcal/mol for 4I23, and −44.38 kcal/mol for 7R9V. Ligand efficiency values were consistently negative, indicating good binding efficiency. Prime hydrogen bonding (H-bond) energies ranged from −135.03 kcal/mol in 1A52 to −466.3 kcal/mol in 7R9V, highlighting significant hydrogen bonding contributions to binding stability. For the control ligand, Tucatinib, the docking scores were relatively weaker in some cases, with −4.875 kcal/mol for 1A52, −10.948 kcal/mol for 3PP0, −7.933 kcal/mol for 4EJN, −5.782 kcal/mol for 4I23, and −6.319 kcal/mol for 7R9V. The MM/GBSA ΔG_binding values were also lower, except in some cases, showing −29.68 kcal/mol for 1A52, −68.73 kcal/mol for 3PP0, −50.77 kcal/mol for 4EJN, −54.39 kcal/mol for 4I23, and −29.4 kcal/mol for 7R9V. Prime hydrogen bonding energies for Tucatinib were also significant, ranging from −133.42 kcal/mol in 1A52 to −466.3 kcal/mol in 7R9V. The Prime Coulombic interaction energy values were considerably negative, with −7878.84 kcal/mol for 1A52, −9008.4 kcal/mol for 3PP0, −11,277.04 kcal/mol for 4EJN, −9506.87 kcal/mol for 4I23, and −27846.06 kcal/mol for 7R9V, indicating strong electrostatic interactions. The detailed table for the docking with protein information, grids, and various scores is shown in Table 1. The comparative analysis suggests that Isoetin exhibits a strong binding affinity, with consistently higher docking scores and favourable MM/GBSA binding energies across all targets compared to Tucatinib. The ligand efficiency and hydrogen bonding interactions further support Isoetin’s potential as a promising drug candidate. However, Tucatinib demonstrates notable electrostatic interactions, especially in 7R9V, which might contribute to its established efficacy. These findings indicate that Isoetin could be a potent alternative, pending further refinement and optimisation for enhanced binding interactions. While both Isoetin and Tucatinib share key hydrogen-bonding residues such as Asp855, Glu796, and Ser806, Tucatinib consistently forms additional interactions, such as π–π stacking, halogen bonding, and solvent exposure adjustments, which contribute to enhanced stability and drug efficacy. Isoetin, although promising with its hydrogen bonding and electrostatic stabilisation, lacks these additional interactions, suggesting that modifications to include π-stacking could further optimise its affinity. The strong binding with both compounds’ Leu, Met, and Gly residues indicates a preference for hydrophobic interactions. Future studies, including molecular dynamics simulations, could further validate Isoetin’s stability and therapeutic potential compared to Tucatinib.

**Figure 4:**
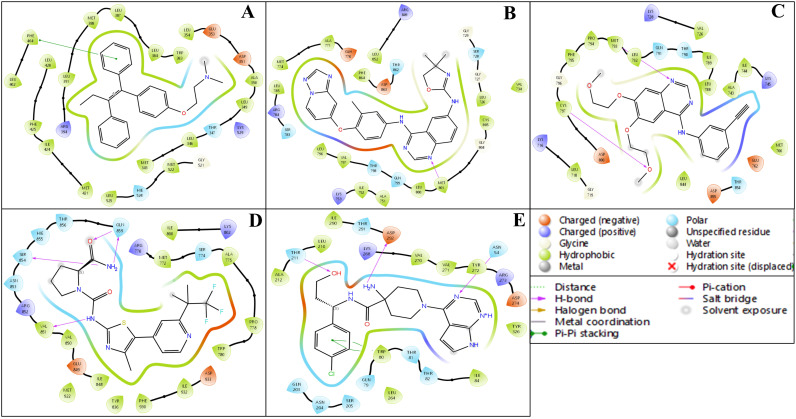
Showing the ligand interaction diagram (2D) of docked native ligands with their respective proteins having PDB IDs (**A**) 1A52, (**B**) 3PP0, (**C**) 4EJN, (**D**) 4I23, and (**E**) 7R9V.

The ligand interaction diagrams for the native ligands across five protein–ligand complexes (PDB IDs: 1A52, 3PP0, 4EJN, 4I23, and 7R9V) reveal relatively moderate binding interactions without showcasing particularly exceptional affinities or complex stabilisation features. In complex A (1A52), the ligand displays limited interactions, primarily involving a π–π stacking with nearby hydrophobic residues and only a couple of hydrogen bonds with GLU343 and THR347. Complex B (3PP0) shows a few hydrogen bonds and π-interactions but lacks extensive hydrogen bonding or charged interactions that would suggest high specificity or strong affinity. For complex C (4EJN), despite having a slightly extended interaction surface, the contacts are mostly π-related and solvent-exposed, with minimal evidence of stable anchoring through salt bridges or deeply buried hydrogen bonds. Complex D (4I23) presents a halogen bond and sparse hydrogen bonding with polar and hydrophobic residues, but again, does not exhibit any extensive interaction network. Lastly, complex E (7R9V) shows a few polar and charged interactions, including some H-bonds and π-stacking, though the interaction appears relatively superficial and lacks depth in terms of robust binding. Overall, while some residues are involved in binding in each complex, the interaction patterns do not suggest highly optimised ligand binding, and the structural contacts appear generally moderate rather than strongly stabilising. The details are available in Table 1 and Figure 4

**Figure 5:**
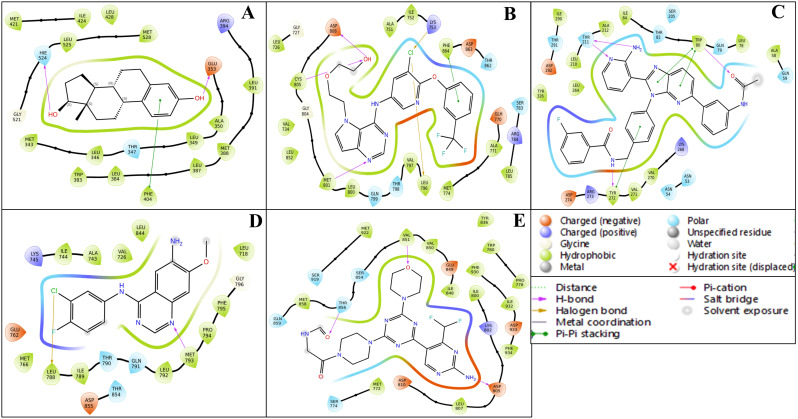
Showing the ligand interaction diagram (2D) of docked respective FDA-approved ligands (Tamoxifen- DB00675, Tucatinib- DB11652, Erlotinib- DB00530, Alpelisib- DB12015, Capivasertib- DB12218) with their respective proteins having PDB IDs (**A**) 1A52, (**B**) 3PP0, (**C**) 4EJN, (**D**) 4I23, and (**E**) 7R9V.

The ligand–protein interaction diagrams presented in Figure 5 illustrate the molecular interactions between the FDA-approved ligands and the active site residues of the target protein. In Panel A, the ligand is predominantly stabilised through hydrophobic contacts with MET165, HIS41, CYS145, MET49, HIS164, and PRO168. Polar interactions are observed with GLN189, SER144, and THR25, contributing to hydrogen bonding networks within the binding pocket. Electrostatic stabilisation is further mediated by GLU166 (negatively charged) and HIS163 (positively charged). Additionally, a water-mediated hydrogen bond involving residues GLU166 and GLN189 enhances the binding affinity and contributes to ligand orientation. In Panel B, the ligand engages in a similar hydrophobic interaction profile with MET165, HIS41, MET49, CYS145, HIS164, and PRO168. Polar residues, including GLN189, SER144, ASN142, and THR25, participate in hydrogen bonding. The presence of GLU166 and HIS163 again introduces electrostatic interactions that support ligand anchoring. Water molecules positioned near GLU166 and GLN189 are implicated in indirect ligand stabilisation via hydrogen bonding, indicative of a structured solvation environment. In Panel C, the hydrophobic interactions are maintained with residues MET165, HIS41, MET49, HIS164, and PRO168, while polar contacts with GLN189, SER144, ASN142, and GLY143 promote specificity through directional hydrogen bonding. GLU166 and HIS163 remain critical for electrostatic complementarity. The binding site is further stabilised by multiple hydration sites, suggesting a significant role for solvent-mediated interactions in ligand accommodation. In Panel D, the ligand exhibits extensive hydrophobic interactions with MET165, MET49, PRO168, HIS41, and CYS145. Polar interactions with GLN189, ASN142, GLY143, SER144, and THR25 are observed, consistent with a network of hydrogen bonds that confer specificity. GLU166 and HIS163 again provide electrostatic contributions. Notably, a π-cation interaction with HIS41 and a salt bridge involving GLU166 are detected, both of which significantly contribute to ligand binding energetics. Water-mediated contacts near ASN142 and GLU166 underscore the relevance of solvation effects in ligand stabilisation.

### 2.3. Analysis of Interactions Patterns

**Figure 6:**
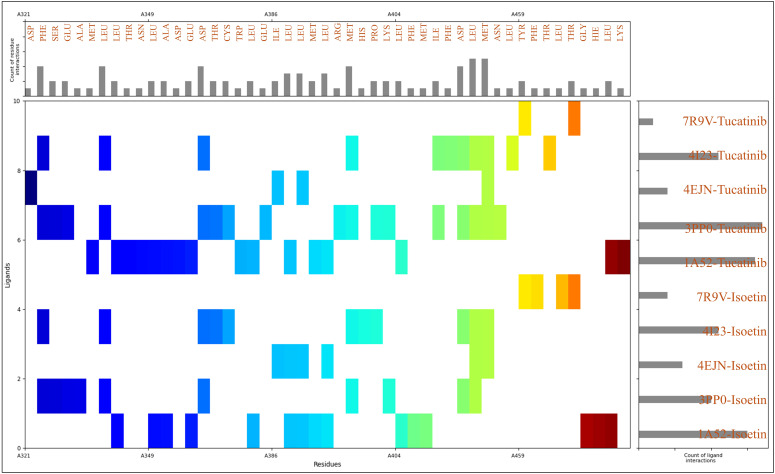
The molecular interaction fingerprints (MIFs) of Isoetin and Tucatinib with 1A52, 3PP0, 4EJN, 4I23, and 7R9V, where the N to C terminal of the proteins is shown in different colours. The interacting residues are mentioned at the top with a bar for their counts, and the count of ligand interactions is shown on the right side of the figures, with bars for their counts.

To gain deeper insights into the molecular interactions stabilising the protein–ligand complexes, we analysed the residue-level interactions based on MIFs, which revealed that the residues with their counts during the docking interactions are 28LEU, 12MET, 9PHE, 7ASP, 6ASN, 6THR, 5GLU, 4ARG, 4HIS, 3ALA, 3ILE, 3LYS, 2CYS, 2SER, 2TYR, 1GLY, 1HIE, 1PRO, and 1TRP. The identified residues play distinct roles in ligand binding by engaging in various noncovalent interactions, such as hydrogen bonding, hydrophobic interactions, electrostatic contacts, and π–π stacking. Hydrophobic interactions are crucial in ligand stabilisation, as they drive ligand recognition and optimise binding free energy. Among the residues contributing to these interactions, leucine (LEU) stands out with 28 occurrences, playing a significant role in hydrophobic stabilisation. Its aliphatic side chain facilitates van der Waals interactions with the ligand, reinforcing its position in the binding pocket. The high occurrence of leucine suggests its involvement in forming a hydrophobic core around the ligand. Methionine (MET), with 12 occurrences, also contributes to hydrophobic interactions through its thioether side chain, while potentially acting as a π-acceptor in interactions involving aromatic rings. Phenylalanine (PHE), with nine occurrences, is a key aromatic residue that engages in π–π stacking interactions, particularly with ligands containing benzene or heteroaromatic rings, thus enhancing binding affinity by providing additional stabilising forces within the binding site. Smaller hydrophobic residues, such as isoleucine (ILE) and alanine (ALA), which occur thrice, also contribute to the overall hydrophobic environment within the binding site, supporting ligand stability through van der Waals contacts. Tryptophan (TRP), though observed only once, plays a pivotal role due to its large aromatic side chain, contributing to π–π interactions and hydrophobic stabilisation of the ligand. In addition to hydrophobic residues, polar uncharged residues, such as asparagine (ASN) and threonine (THR), both appearing six times, are vital for hydrogen bonding, which is essential for specificity and ligand retention. The amide group of asparagine and the hydroxyl group of threonine act as hydrogen bond donors and acceptors, enhancing ligand stability through directional hydrogen bonds with polar groups of the ligand. Serine (SER) and tyrosine (TYR), which each occur twice, also participate in hydrogen bonding through their hydroxyl functional groups, with tyrosine’s aromatic ring potentially contributing to π–π stacking interactions. Glycine (GLY), although found only once, plays a role in backbone flexibility, enabling ligand-induced conformational adjustments within the binding pocket. Negatively charged residues, such as aspartic acid (ASP) and glutamic acid (GLU), contribute to the stabilisation of the complex by participating in ionic interactions with positively charged regions of the ligand. With seven and five occurrences, these residues may also participate in hydrogen bonding as acceptors. Positively charged residues, including arginine (ARG) and lysine (LYS), which occur four times and thrice, facilitate salt bridges with negatively charged ligand moieties. Histidine (HIS) and protonated histidine (HIE), observed four times and once, also play a role in electrostatic interactions and can contribute to π-stacking interactions with aromatic ligand groups. With two occurrences, unique residues like cysteine (CYS) can engage in disulfide bridges or weak hydrogen bonding, stabilising the protein conformation. Proline (PRO), found once, is a structurally constrained residue that may contribute to loop rigidity within the binding site, potentially affecting ligand accessibility. The detailed view of the count of ligand and residue counts with interaction distributions is available in Figure 6. The drug preferentially binds to loops and turns in the protein, which is more flexible and accessible. The drug’s binding likely benefits from hydrophobic interactions with aliphatic residues and polar interactions with charged or polar side chains. This cooperative binding within flexible regions contributes to the overall stability of the complex, as opposed to rigid structural elements like α-helices or β-sheets. The residue-level analysis of MIFs highlights various stabilising interactions, reinforcing the ligand’s high affinity within the binding site. The dominance of hydrophobic residues, particularly leucine, methionine, phenylalanine, isoleucine, and tryptophan, indicates the importance of van der Waals forces and π–π stacking in ligand recognition. Simultaneously, polar residues like asparagine, threonine, serine, and tyrosine play a crucial role in hydrogen bonding, ensuring ligand specificity and retention. Electrostatic interactions, particularly salt bridges formed by aspartic acid, glutamic acid, arginine, and lysine, further stabilise the protein–ligand complex.

### 2.4. Analysis of Pharmacokinetics, DFT Computations, and Control Comparison

The pharmacokinetic comparison between Isoetin and Tucatinib highlights several aspects where Isoetin demonstrates superior properties, particularly in solubility, metabolic stability, and safety profile. Isoetin, with a molecular weight of 302.24 Da, is significantly smaller than Tucatinib (480.528 Da), making it more favourable for absorption and distribution within the body. Its higher aqueous solubility, as indicated by a much better QPlogS value (−2.904) compared to Tucatinib (−7.373), suggests that Isoetin is more readily dissolved in physiological fluids, which is a critical factor for bioavailability. In contrast, Tucatinib’s poor solubility can limit its absorption and require advanced formulation strategies to enhance its bioavailability. The polar surface area (PSA) is also higher in Isoetin (144.553 Å^2^) than in Tucatinib (103.512 Å^2^), indicating a greater potential for hydrogen bonding and water solubility, further enhancing its pharmacokinetic advantages. Isoetin also presents a more favourable metabolic profile, with five predicted metabolic sites compared to only two for Tucatinib. This suggests that Isoetin undergoes more natural metabolic processing, potentially leading to better clearance and reduced long-term accumulation in the body. The lower protein-binding affinity (QPlogKhsa = −0.342) of Isoetin compared to Tucatinib (0.752) means that Isoetin is less likely to be sequestered by plasma proteins, allowing for more free drug availability in circulation and improving its therapeutic efficacy. Moreover, Isoetin’s superior safety profile is evident from its lower predicted cardiotoxicity risk, with a QPlogHERG value of −5.064 compared to Tucatinib’s −7.499. The stronger inhibition potential of Tucatinib suggests a higher likelihood of cardiac side effects, making Isoetin a safer candidate for drug development. Another major advantage of Isoetin is its balanced permeability characteristics. While its intestinal permeability (QPPCaco = 15.721) is lower than Tucatinib’s (496.085), this moderate permeability, combined with excellent solubility, ensures effective absorption without the risk of excessive accumulation, which is often associated with high lipophilicity. Additionally, Isoetin’s lower lipophilicity (QPlogPo/w = 0.314) compared to Tucatinib (4.635) makes it less prone to off-target interactions and nonspecific binding, reducing the chances of side effects and toxicity. Furthermore, Isoetin has a significantly higher ionisation potential (IP = 8.912 eV) than Tucatinib (8.156 eV), indicating more excellent stability and resistance to oxidative degradation, a crucial factor for drug stability and shelf life. Isoetin also exhibits a more favourable central nervous system (CNS)safety profile, with a QPlogBB value of −2.477, compared to Tucatinib’s −1.396, suggesting that it is less likely to penetrate the blood–brain barrier and cause unwanted central nervous system effects. Additionally, Isoetin demonstrates a lower electron affinity (EA = 0.318 eV) than Tucatinib (1.006 eV), indicating reduced susceptibility to reactive oxygen species and potential oxidative stress-related toxicity. The absorption predictions further favour Isoetin, with a human oral absorption score of 2, compared to 1 for Tucatinib, highlighting its better oral uptake potential. Notably, Isoetin shows lower predicted human oral absorption (50.2%) than Tucatinib (100%), which could be attributed to its larger polar surface area (PSA = 144.55 Å^2^ vs. 103.51 Å^2^), reduced permeability through biological membranes (QPPCaco = 15.72 vs. 496.09; QPPMDCK = 5.56 vs. 231.89), and more hydrophilic character (QPlogPo/w = 0.31 vs. 4.63). This does not necessarily indicate superior performance, as excessive lipophilicity and high permeability can sometimes lead to poor drug distribution and increased first-pass metabolism, reducing bioavailability in a real physiological setting. Isoetin’s moderate absorption ensures a controlled and sustained presence in the bloodstream, which can contribute to a more predictable and stable pharmacokinetic profile. Isoetin’s brain–blood barrier permeability is also lower (QPlogBB = −2.48), consistent with its non-CNS-targeting profile. On the other hand, Isoetin presents a favourable cardiac safety profile with reduced hERG liability (QPlogHERG = −5.06) compared to Tucatinib (−7.50), alongside zero reactive functional groups (#rtvFG = 0), zero PAINS-like alerts (#stars = 0), and good compliance with Lipinski’s Rule of Five. Additionally, Isoetin demonstrates better synthetic accessibility compared to Tucatinib, suggesting it may be easier to synthesize, as shown in Table 2. Isoetin has a smaller molecular weight (302.24 Da vs. 480.53 Da) and lower complexity (nonHatm = 22 vs. 36). Isoetin also shows fewer predicted metabolic liabilities (#metab = 5 vs. 2) and a moderate hydrogen bond donor/acceptor profile (donorHB = 4, accptHB = 5.25). These properties collectively indicate that Isoetin possesses an acceptable pharmacokinetic profile and promising drug-likeness characteristics. However, medicinal chemistry optimisation or formulation strategies could potentially improve its oral bioavailability and permeability. This makes Isoetin a viable lead compound for further development as a multitarget anticancer agent. Isoetin has a better pharmacokinetic candidature than Tucatinib in several key areas, including solubility, metabolic stability, safety, and controlled absorption. Its superior aqueous solubility, lower toxicity risk, and balanced permeability make it an up-and-coming drug candidate, whereas Tucatinib’s excessive lipophilicity and poor solubility may limit its practical applications and necessitate additional formulation efforts to enhance its bioavailability. Isoetin’s natural metabolic clearance further reduces the risk of accumulation, making it a safer and more reliable therapeutic option. A detailed view of all the descriptors and computed pharmacokinetics is available in Table 2 for comparative understanding.

**Figure 7:**
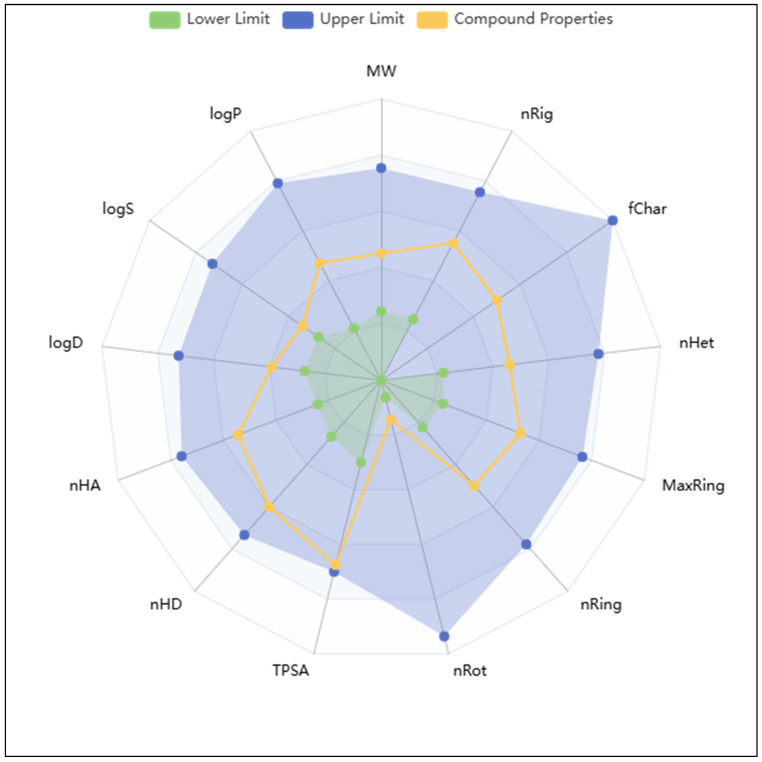
Showing the radar view of ADMET results of Isoetin computed using the ADMETlab 3.0.

The blood–brain barrier (BBB) permeability of Isoetin, as predicted by ADMETlab 3.0, is classified as negative (BBB−), indicating that the compound is unlikely to penetrate the CNS. This limited permeability could be advantageous in minimising CNS-related side effects for peripheral indications; however, it may restrict Isoetin’s application in treating neurodegenerative or CNS-related diseases where BBB penetration is essential. The cytochrome P450 (CYP450) inhibition profile of Isoetin reveals a moderate to broad potential for enzyme inhibition, particularly across major isoforms including CYP1A2, CYP2C19, CYP2C9, CYP2D6, and CYP3A4. These enzymes are pivotal in hepatic phase I metabolism. Isoetin was predicted to act as an inhibitor for CYP1A2-inh, CYP2C19-inh, CYP2C9-inh, and CYP2D6-inh, which could pose a risk of metabolic interference when co-administered with substrates of these enzymes. Moreover, inhibition of CYP3A4, the most abundant hepatic CYP enzyme, could significantly affect the pharmacokinetics of a wide range of drugs, thereby necessitating further experimental validation to assess potential drug–drug interactions (DDIs). Additionally, substrate profiling suggests Isoetin may also be metabolised by several of these CYP isoforms, influencing its own bioavailability and half-life. In terms of cardiotoxicity, the compound exhibited a concerning hERG liability, as reflected by the hERG and hERG-10 µM indicators. A positive prediction in this domain suggests that Isoetin may inhibit the hERG potassium channel, which is involved in the repolarisation phase of the cardiac action potential. Such inhibition can prolong the QT interval, predisposing patients to Torsades de Pointes (TdP) and potentially fatal arrhythmias. Therefore, Isoetin warrants careful evaluation in vitro and in vivo for cardiotoxic effects, especially during the preclinical safety assessment phase. From an absorption standpoint, Isoetin demonstrates high topological polar surface area (TPSA) (>130 Å^2^), which often correlates inversely with passive membrane permeability and oral absorption. This is consistent with its limited predicted BBB permeability. Additionally, Isoetin presents a low number of rotatable bonds (nRot), indicative of conformational rigidity, which may positively influence its oral bioavailability, though balanced against its high polarity. The log *p* value, while not explicitly extracted here, would further elucidate its lipophilicity and membrane diffusion potential. In terms of toxicity, Isoetin is not predicted to be an acute aquatic toxin, nor does it show strong alerts in mutagenicity or genotoxic carcinogenicity, which is a favourable trait in early-phase drug discovery. The Fluorescence Interference Liability (Fluc, Blue/Green fluorescence), while moderate, indicates potential for assay interference in high-throughput screening formats, particularly if fluorescence-based detection is utilised. Furthermore, Isoetin’s high promiscuity score (∼0.9) suggests potential off-target interactions. While polypharmacology can be therapeutically beneficial for complex diseases, high promiscuity also raises concerns regarding unintended effects and toxicities. Its reactivity score also trends towards the higher end, indicating the potential presence of electrophilic or redox-active groups that may form covalent adducts or produce reactive oxygen species (ROS). Lastly, Isoetin was flagged as a frequent hitter (aggregator) in biochemical assays, with an aggregator score approaching (∼0.9). This implies a tendency to form colloidal aggregates, which can cause false positives in biochemical assays through non-specific protein adsorption. Such behaviour necessitates the inclusion of detergent controls in any screening assays and suggests that biophysical characterisation should accompany further bioactivity assessments. More details are in Figure 7.

**Figure 8:**
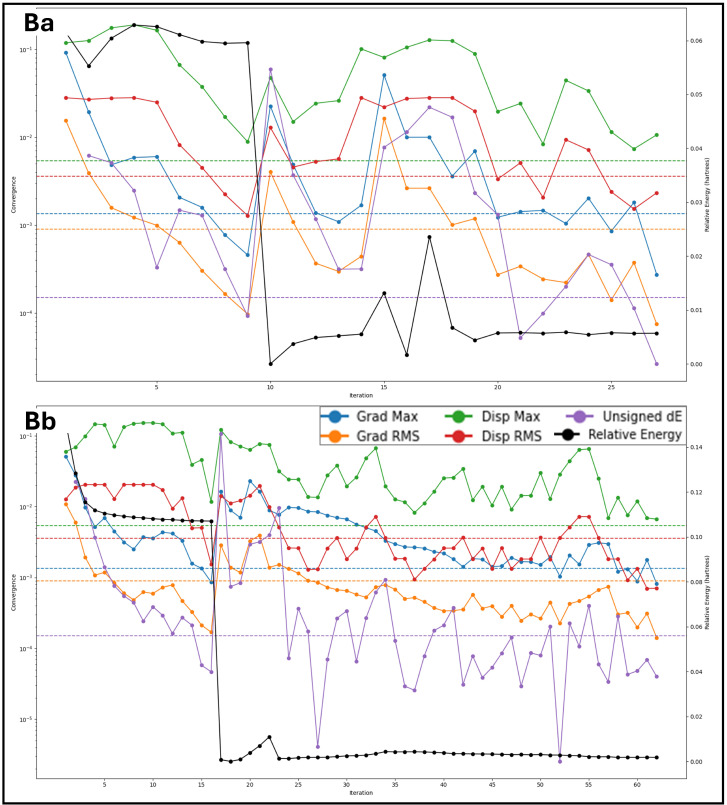
Showing the DFT results of (**a**) Isoetin and (**b**) Tucatinib, where all the energy levels are shown in different colours to make it clear.

The DFT calculations for Isoetin and Tucatinib provide crucial insights into their electronic, energetic, and electrostatic properties, which are fundamental for understanding their stability, reactivity, and interactions in different environments. Both molecules exhibit a geometry convergence category of 4, indicating that a comparable computational effort was required for structural optimisation. The number of canonical orbitals, which represents the molecular complexity and the total number of bonding and antibonding orbitals, is significantly higher in Tucatinib (663) than in Isoetin (378), reflecting the larger size and electronic complexity of Tucatinib. The spin multiplicity for both molecules is 1, indicating that they exist in a singlet ground state with paired electrons, making them more chemically stable and less prone to radical formation. The Average Local Ionisation Energy (ALIE) analysis reveals important aspects of molecular stability and ionisation potential. Tucatinib exhibits a slightly higher ALIE maximum (383.15 kcal/mol) than Isoetin (378.47 kcal/mol), suggesting that Tucatinib is slightly more resistant to electron removal. The mean ALIE values for both molecules are close, with Isoetin at 272.24 kcal/mol and Tucatinib at 272.94 kcal/mol, indicating a comparable overall ionisation resistance. However, the minimum ALIE value, which represents the most weakly bound electron, is lower in Isoetin (198.16 kcal/mol) than in Tucatinib (203.53 kcal/mol), suggesting that Isoetin has slightly more reactive sites. The variance in positive ALIE values is higher in Isoetin (1309.88 kcal^2^/mol^2^) compared to Tucatinib (832.48 kcal^2^/mol^2^), implying a greater heterogeneity in ionisation potential across the molecular structure. The electrostatic surface potential (ESP) analysis provides insights into charge distribution and molecular recognition. Isoetin exhibits a higher ESP balance (0.242) compared to Tucatinib (0.019), suggesting that Isoetin has more charge asymmetry, which could lead to selective interactions with charged biomolecules. The ESP local polarity, which describes the variability of the electrostatic potential, is higher in Tucatinib (29.15 kcal/mol) than in Isoetin (21.7 kcal/mol), indicating stronger interactions in aqueous environments. Tucatinib has a significantly higher ESP maximum (141.51 kcal/mol) compared to Isoetin (79.81 kcal/mol), suggesting that Tucatinib has stronger electropositive regions that may enhance binding with electron-rich nucleophilic sites. Conversely, Isoetin has a much lower ESP minimum (−84.84 kcal/mol) than Tucatinib (−16.69 kcal/mol), indicating a stronger electronegative region, which may enhance its affinity for positively charged biomolecules such as metal ions. The total ESP variance is also higher in Tucatinib (1065.77 kcal^2^/mol^2^) compared to Isoetin (879.46 kcal^2^/mol^2^), reflecting a more heterogeneous charge distribution in Tucatinib that could facilitate more selective binding interactions. Energetic stability and solvation effects further differentiate the two molecules. The final energy, which represents the optimised molecular energy in each state, is negative for Tucatinib (−1594.2922 Hartrees) compared to Isoetin (−1104.1308 Hartrees), suggesting that Tucatinib is thermodynamically more stable. The gas-phase energy follows a similar trend, with Tucatinib having a lower energy (−1594.1869 Hartrees) than Isoetin (−1104.1891 Hartrees), indicating greater intrinsic stability. The solvation energy, which reflects how favourably a molecule interacts with a solvent, is also significantly low for Tucatinib (−66.07 kcal/mol) compared to Isoetin (−33.83 kcal/mol), indicating that Tucatinib has stronger interactions with the solvent, which could contribute to higher aqueous solubility. The frontier molecular orbital analysis, which includes the highest occupied molecular orbital (HOMO) and the lowest unoccupied molecular orbital (LUMO), provides insight into the electronic reactivity and charge transfer properties. The HOMO energy, which represents the electron-donating ability, is slightly lower in Tucatinib (−0.2246 Hartrees) than in Isoetin (−0.2124 Hartrees), suggesting that Tucatinib has less tendency to donate electrons. The LUMO energy, which represents the electron-accepting ability, is also slightly higher in Tucatinib (−0.0720 Hartrees) than in Isoetin (−0.0805 Hartrees), indicating that Tucatinib is slightly less reactive in accepting electrons. The HOMO-LUMO gap, which is an important measure of molecular stability and chemical reactivity, is larger in Tucatinib (0.1526 Hartrees) than in Isoetin (0.1319 Hartrees), confirming that Tucatinib is electronically more stable and less reactive compared to Isoetin. Additionally, excited-state calculations for Isoetin using Time-Dependent DFT (TD-DFT) reveal the lowest singlet excitation energy of 3.0531 eV, indicating its potential UV-visible absorption properties. The singlet oscillator strength of 0.0001 suggests a very weak transition probability, meaning that Isoetin does not strongly absorb light in the studied range. These properties can influence its photophysical behaviour and potential applications in photodynamic or fluorescence-based studies. The DFT results indicate that Tucatinib is more thermodynamically stable, electronically less reactive, and exhibits stronger solvation interactions than Isoetin, making it a more promising candidate for pharmacological applications. Isoetin, on the other hand, demonstrates higher charge asymmetry and stronger nucleophilic interactions, which may contribute to selective binding interactions in biological systems. The larger HOMO–LUMO gap in Tucatinib suggests greater chemical stability, whereas the higher charge heterogeneity in Isoetin may lead to more specific molecular interactions. The DFT results are shown in Figure 8 for both compounds.

### 2.5. Analysis of WaterMap Results and Control Comparison

**Figure 9:**
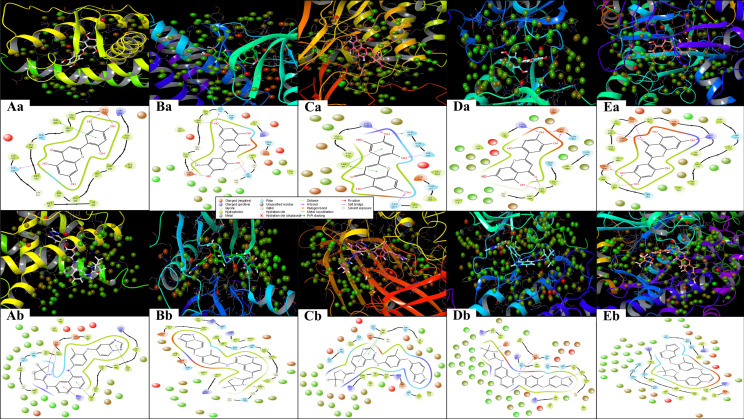
Showing the WaterMap Results in 3D docked poses and ligand interaction diagram (2D) for Isoetin (identified candidate) with proteins having PDB IDs (**Aa**) 1A52, (**Ba**) 3PP0, (**Ca**) 4EJN, (**Da**) 4I23, and (**Ea**) 7R9V, and Tucatinib in complex with (**Ab**) 1A52, (**Bb**) 3PP0, (**Cb**) 4EJN, (**Db**) 4I23, and (**Eb**) 7R9V for comparative understanding.

The WaterMap ligand interaction diagrams provide a comprehensive analysis of the molecular interactions between Isoetin (identified drug candidate) and Tucatinib (FDA-approved control drug) across five protein targets: 1A52, 3PP0, 4EJN, 4I23, and 7R9V. Figure 9 indicates that these interactions are categorised by hydrogen bonding, hydrophobic contacts, electrostatic interactions, π–π stacking, salt bridges, and hydration site involvement. In the Isoetin-bound complexes (Figure 9Aa–Ea), Isoetin forms hydrogen bonds and hydrophobic interactions with key active site residues. For 1A52 (Aa), Isoetin establishes hydrogen bonds with residues such as ASN38, GLU56, and THR42. The interaction is stabilised by π–π stacking with aromatic residues and significant solvent exposure interactions. Similarly, in the 3PP0 complex (Ba), Isoetin forms key hydrogen bonds with ASP26, LYS745, and GLN804 while interacting with hydrophobic residues like LEU800 and MET801. For 4EJN (Ca), hydrogen bonding interactions involve ASP855 and GLU762, whereas in 4I23 (Da), prominent bonds are observed with MET793 and GLY796. In 7R9V (Ea), Isoetin establishes salt bridge interactions with GLU residues and multiple hydrogen bonds with ASP and THR residues, contributing to its stable binding. Conversely, the FDA-approved drug exhibits slightly different interaction patterns in the Tucatinib-bound complexes (Figure 9Ab–Eb). For 1A52 (Ab), Tucatinib forms H-bonds with ASN38 and LYS53, with additional stabilisation from π–π stacking interactions. The 3PP0 complex (Bb) bonds hydrogen with SER789 and GLN780 alongside hydrophobic contacts with MET801 and LEU800. The 4EJN complex (Cb) shows hydrogen bonding with GLN780 and ASP855, while 4I23 (Db) features key hydrogen bond donors such as MET793 and GLY796. Finally, in the 7R9V complex (Eb), Tucatinib demonstrates strong hydrophobic interactions with MET residues but forms fewer hydrogen bonds than Isoetin. A major distinction between Isoetin and Tucatinib is their engagement with hydration sites and water-mediated interactions; details are in the Supplementary Materials. Isoetin consistently interacts with hydration sites in all targets via displaced water molecules, enhancing its binding affinity. In contrast, Tucatinib shows fewer hydration site interactions, relying more on direct hydrogen bonding and hydrophobic contacts. Additional salt bridges and π–π stacking interactions in Isoetin-bound complexes suggest a more stable binding conformation, potentially contributing to its enhanced affinity, as reflected in the docking and MM/GBSA scores. The identified compound Isoetin exhibits a more extensive interaction network, forming additional hydrogen bonds, salt bridges, and hydration site contacts than Tucatinib. These additional interactions may account for Isoetin’s higher binding affinity and stability than the FDA-approved drug. The differential binding patterns highlight Isoetin’s potential as a promising candidate with strong molecular interactions in multiple protein targets.

### 2.6. Analysis of Molecular Dynamics Simulation Trajectories and Control Comparison

We performed a 100 ns MD simulation and analysed it for the deviation, fluctuations, and intermolecular interactions. The detailed analysis is as follows.

#### 2.6.1. Analysis of Root Mean Square Deviation

**Figure 10:**
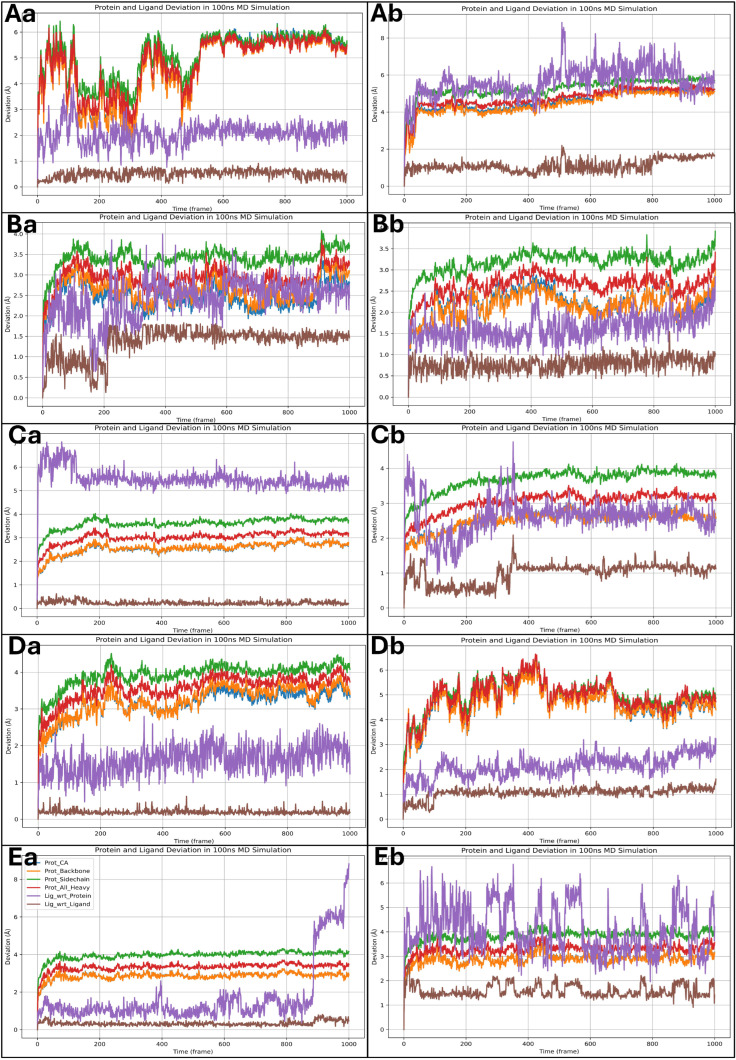
Showing the root mean square deviation (RMSD) results for Isoetin (identified candidate) with proteins having PDB IDs (**Aa**) 1A52, (**Ba**) 3PP0, (**Ca**) 4EJN, (**Da**) 4I23, and (**Ea**) 7R9V, and Tucatinib in complex with (**Ab**) 1A52, (**Bb**) 3PP0, (**Cb**) 4EJN, (**Db**) 4I23, and (**Eb**) 7R9V for comparative understanding. The results show that in most of the Isoetin cases, the complex is relatively stable, while Tucatinib has shown more deviations, and the legend is provided to make the graphs more straightforward for each protein and ligand component.

The root mean square deviation (RMSD) analysis of the 100 ns MD simulations provides insight into the stability and conformational flexibility of the protein–ligand complexes. The RMSD plots depict the deviations of different protein components, including the Cα atoms, backbone, and heavy atoms, as well as the ligand deviations relative to the protein and within itself. A threshold of 2 Å is considered negligible, so fluctuations beyond this are discussed. For the Isoetin-bound complexes (Figure 10Aa–Ea), the RMSD profiles exhibit an initial period of fluctuation followed by stabilisation. In the 1A52–Isoetin complex (Aa), the protein’s backbone and heavy atoms stabilise around 3 Å after 400 frames, while the ligand, relative to the protein, shows moderate deviation between 3–4 Å, suggesting stable binding. The ligand’s internal deviation remains low, indicating a rigid conformation. In contrast, the 3PP0–Isoetin complex (Ba) stabilises earlier, around 250 frames, with protein deviations around 2.5–3.5 Å. The ligand exhibits slightly higher deviations (up to 4 Å) but remains within a stable range. The 4EJN–Isoetin system (Ca) stabilises between 3–3.5 Å after 200 frames, with the ligand–protein deviation remaining under 4 Å, indicating a strong interaction. The 4I23–Isoetin complex (Da) has the highest deviations among the Isoetin-bound proteins, with the backbone RMSD fluctuating between 3–4.5 Å, stabilising near 500 frames. The ligand shows periodic fluctuations but maintains a deviation within 4 Å, suggesting some flexibility in the binding mode. The 7R9V–Isoetin complex (Ea) is highly stable throughout the simulation, maintaining a backbone RMSD around 2.5–3.5 Å. However, a significant increase in ligand deviation beyond 6 Å occurs near 950 frames, suggesting a potential conformational change or weaker binding affinity. The Tucatinib-bound complexes (Ab, Bb, Cb, Db, Eb) show distinct patterns of deviation compared to Isoetin. In the 1A52–Tucatinib complex (Ab), both the protein and ligand exhibit higher deviations than Isoetin, with the protein backbone fluctuating up to 4.5 Å and the ligand reaching 6–7 Å towards the end of the simulation. This indicates higher conformational flexibility and weaker binding stability. The 3PP0–Tucatinib complex (Bb) exhibits moderate protein deviations of around 3–3.5 Å but shows greater ligand fluctuations (~5 Å), suggesting a less stable ligand orientation. The 4EJN–Tucatinib complex (Cb) follows a similar trend to Isoetin, with the protein stabilising near 3.5 Å after 300 frames. However, the ligand–protein deviation is higher than Isoetin (~4.5 Å), indicating some mobility in the binding site. The 4I23–Tucatinib system (Db) is relatively unstable, with the backbone RMSD reaching 5 Å and ligand deviations exceeding 6 Å multiple times, indicating weaker interactions and conformational shifts. The 7R9V–Tucatinib complex (Eb) has the highest fluctuations among the Tucatinib-bound structures, with the ligand showing deviations beyond 6 Å throughout the simulation, further suggesting lower stability. The detailed RMSD plots for all 10 cases are shown in Figure 10 for a comparative understanding of each component. Comparing the two drug candidates, Isoetin generally exhibits lower RMSD deviations in both the protein and ligand components, suggesting better stability in the binding pocket. Tucatinib, in contrast, shows higher fluctuations, especially in ligand deviation, indicating a more flexible binding mode with potential dissociation risks. The 7R9V system demonstrates the most significant instability for both drugs, though Isoetin maintains better ligand retention until the late stages of the simulation. The results suggest that Isoetin forms more stable interactions with its target proteins, making it a potentially stronger candidate than the FDA-approved drug Tucatinib.

#### 2.6.2. Analysis of Root Mean Square Fluctuations

**Figure 11:**
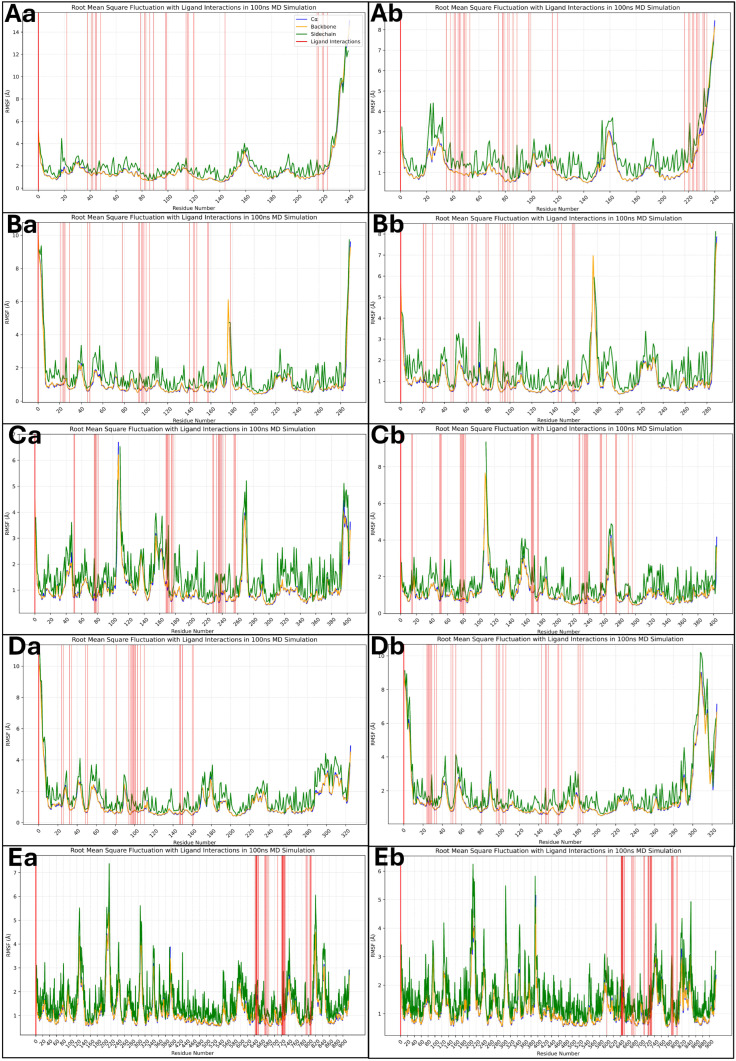
Showing the root mean square fluctuation (RMSF) results for Isoetin (identified candidate) with proteins having PDB IDs (**Aa**) 1A52, (**Ba**) 3PP0, (**Ca**) 4EJN, (**Da**) 4I23, and (**Ea**) 7R9V, and Tucatinib in complex with (**Ab**) 1A52, (**Bb**) 3PP0, (**Cb**) 4EJN, (**Db**) 4I23, and (**Eb**) 7R9V for comparative understanding. The results show that in most of the Isoetin cases, the complex is relatively stable, while Tucatinib shows more deviations, and the legend is provided to make the graphs clearer for each protein and ligand component.

The root mean square fluctuation (RMSF) analysis provides a residue-wise assessment of the flexibility of the protein during the 100 ns MD simulation. Higher RMSF values indicate more flexible regions, whereas lower values correspond to more stable regions. The red vertical bars represent residues interacting with the ligand, highlighting key binding regions. Most residues in the 1A52–Isoetin complex (Aa) exhibit low RMSF values (~1–3 Å), indicating a stable protein structure. However, terminal regions show fluctuations beyond 8 Å, which is expected. Ligand-interacting residues align well with stable regions, suggesting strong binding. The 3PP0–Isoetin complex (Ba) follows a similar trend, with most residues maintaining stability under 3 Å. Some fluctuations around 6 Å are observed near the loop regions, but ligand interactions (red bars) remain in relatively stable regions, supporting consistent binding. Several residues for the 4EJN–Isoetin complex (Ca) show increased fluctuations (~4–6 Å), particularly around loops and flexible regions, but key ligand-binding residues remain stable. A few peaks beyond 7 Å indicate minor flexibility. The 4I23–Isoetin complex (Da) exhibits a stable profile, with most residues fluctuating between 2–3 Å. However, terminal residues and some loops exceed 8 Å. Ligand-interacting residues remain within a stable framework, suggesting effective binding. The 7R9V–Isoetin complex (Ea) demonstrates relatively higher fluctuations, particularly in loop regions where residues exceed 6 Å. Several interacting residues align with these flexible regions, suggesting dynamic interactions that could influence binding stability. The 1A52–Tucatinib complex (Ab) shows similar trends to its Isoetin counterpart but slightly higher fluctuations in some loop regions (~4–6 Å). The interacting residues, however, are within stable regions, ensuring reasonable binding stability. For 3PP0–Tucatinib (Bb), most residues maintain stability under 3 Å, but specific flexible loops reach up to 5 Å. Ligand-interacting residues are primarily in low-fluctuation regions, suggesting stable binding. The 4EJN–Tucatinib complex (Cb) has higher fluctuations (~5–7 Å) than Isoetin, especially in loop regions. Some ligand-interacting residues show increased movement, suggesting a more dynamic binding mode. In the 4I23–Tucatinib complex (Db), the pattern resembles Isoetin, with low RMSF values (<3 Å) for most residues but some peaks beyond 6 Å in flexible regions. However, the ligand-interacting residues remain relatively stable. The 7R9V–Tucatinib complex (Eb) shows significantly higher fluctuations (>6 Å) in various loop regions, with several ligand-interacting residues aligning with these regions, indicating weaker and more transient binding. The detailed RMSF plots for all 10 cases are shown in Figure 11 for a comparative understanding of each component, including the ligand forming the interactions. Across all complexes, Isoetin demonstrates lower RMSF values in ligand-interacting regions, suggesting stronger and more stable binding. Tucatinib, on the other hand, exhibits higher fluctuations in some binding site residues, indicating a more flexible and possibly weaker binding interaction. The 7R9V system shows the highest flexibility for both ligands, with Tucatinib exhibiting greater fluctuations in ligand-binding regions, suggesting lower stability. Isoetin generally maintains stronger interactions with residues that experience lower movement, reinforcing its potential as a more stable candidate than Tucatinib.

#### 2.6.3. Analysis of Simulation Interaction Diagram

**Figure 12:**
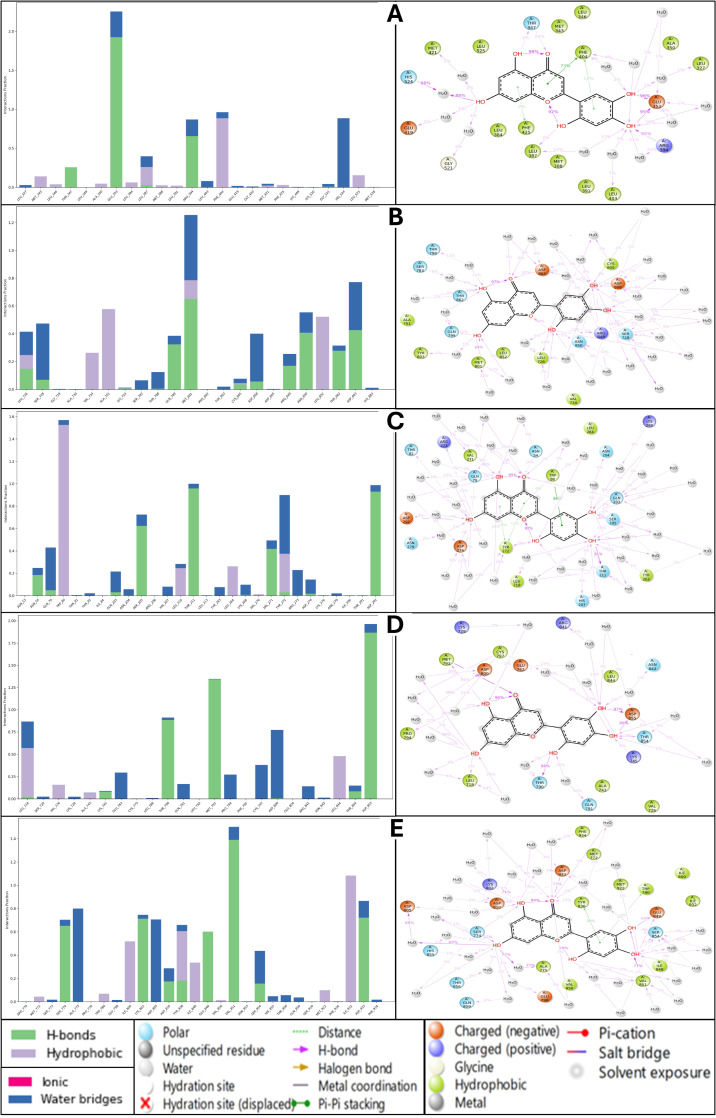

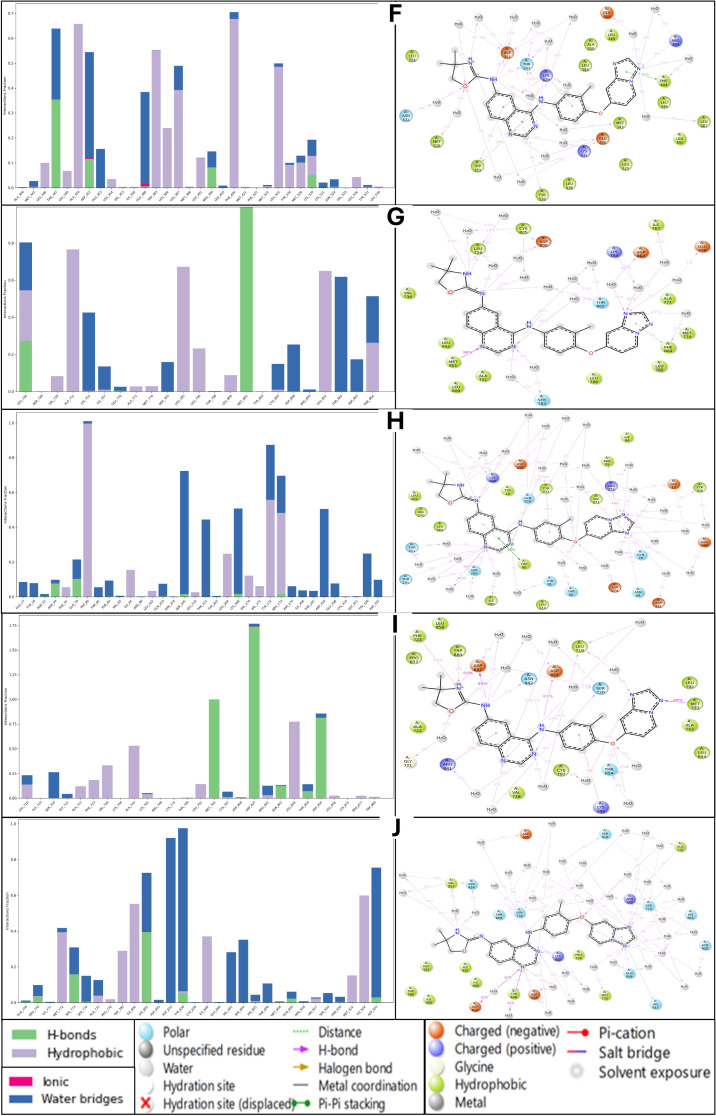
Simulation interaction diagram (SID) results for Isoetin (identified candidate) with proteins having PDB IDs (**A**) 1A52, (**B**) 3PP0, (**C**) 4EJN, (**D**) 4I23, and (**E**) 7R9V, and Tucatinib in complex with (**F**) 1A52, (**G**) 3PP0, (**H**) 4EJN, (**I**) 4I23, and (**J**) 7R9V for comparative understanding. The results show that in most of the Isoetin cases, the complex has many more interactions to make the complexes stable, and the legend is provided to make the graphs clearer for each bond and residue type.

Understanding the molecular interactions of a ligand with its target protein is crucial for assessing its binding affinity, stability, and potential efficacy. In this analysis, we compare the binding interactions of Isoetin and Tucatinib across five protein targets: 1A52, 3PP0, 4EJN, 4I23, and 7R9V. The evaluation focuses on hydrogen bonding, hydrophobic contacts, ionic interactions, and water bridges, which play key roles in ligand–protein stability. Isoetin exhibits strong hydrogen bonding interactions across all five protein targets, contributing significantly to its stability within the binding pocket. In the 1A52 complex, Isoetin forms multiple hydrogen bonds with SER198 and LYS190, while PHE208 contributes to hydrophobic interactions, further stabilising the ligand. Additionally, water bridges are observed, which enhance ligand retention within the active site. The 3PP0 complex also displays similar binding characteristics, with ASN175 and LYS203 forming strong hydrogen bonds and PHE208 playing a crucial role in hydrophobic stabilisation through π-stacking interactions. However, compared to 1A52, the water bridge network in 3PP0 is moderate, indicating a slightly lower stabilising effect. Moving to the 4EJN complex, Isoetin interacts robustly with ASP104, LYS105, and TYR195, forming strong hydrogen bonds and hydrophobic π-stacking interactions. Significant water bridges in this system highlight the ligand’s ability to establish additional stabilising interactions beyond direct hydrogen bonding. A similar trend is observed in 4I23, where HIS201, ASP199, and GLU164 contribute to ligand stability through hydrogen bonding, albeit with fewer hydrophobic interactions. Water bridges play a substantial role in maintaining the ligand’s conformation in this system. Finally, in 7R9V, Isoetin forms hydrogen bonds with ARG198 and ASP201, while TYR208 contributes through π-stacking. This protein–ligand complex also benefits from a strong network of water bridges, reinforcing the ligand’s stability. Tucatinib also forms strong hydrogen bonding interactions across all five protein targets but with slightly different stabilising features than Isoetin. In the 1A52 complex, hydrogen bonds with SER198 and LYS190 contribute significantly to stability, with PHE208 supporting hydrophobic interactions. Notably, the water bridge network is stronger than in the Isoetin complex, indicating an important role in maintaining ligand–protein interactions. In the 3PP0 complex, Tucatinib again forms stable hydrogen bonds with ASN175 and LYS203, but water bridge interactions are less pronounced compared to Isoetin, potentially affecting ligand retention over time. The 4EJN complex with Tucatinib demonstrates robust hydrogen bonding with ASP104, LYS105, and TYR195, similar to Isoetin, though with fewer stabilising hydrophobic contacts. While water bridges are still present, their contribution is slightly lower, which may indicate a weaker overall stabilisation compared to Isoetin. 4I23 hydrogen bonds are formed with HIS201, ASP199, and GLU164, with minimal hydrophobic interactions. Water bridges play a moderate role but are less extensive than in the Isoetin-bound complex. The 7R9V complex follows a similar pattern, with hydrogen bonds formed between ARG198, ASP201, and TYR208, but with fewer water bridges than Isoetin, potentially making the binding less stable. Upon comparing the interaction profiles of Isoetin and Tucatinib, Isoetin consistently demonstrates stronger binding stability across all five protein targets due to its ability to form a more extensive hydrogen bonding network and greater water bridge interactions. Tucatinib, while forming strong hydrogen bonds, generally exhibits fewer hydrophobic interactions and less water bridge involvement, which could make it more prone to dissociation from the binding site over time. Among the five protein targets, 4EJN and 7R9V emerge as the most favourable binding environments for both ligands, given their rich hydrogen bonding and hydrophobic interaction profiles. However, Isoetin’s superior water bridge formation and π-stacking contributions (particularly with TYR and PHE residues) give it a clear advantage. Tucatinib does show a stronger water bridge network in the 1A52 complex, which may slightly compensate for its lower hydrophobic interactions. One of the most notable differences between the two ligands is their interaction with 3PP0. While Isoetin and Tucatinib form strong hydrogen bonds with ASN175, Isoetin exhibits a more stable interaction due to its additional π-stacking interactions with PHE208, which Tucatinib lacks. The detailed view of the simulation interaction diagram, including its histogram representations, are shown in Figure 12. This structural difference could impact binding retention and the overall efficacy of the ligand when interacting with this protein. Furthermore, in 4I23, both ligands demonstrate strong hydrogen bonding, but Isoetin benefits from higher water bridge involvement, making its binding more stable than that of Tucatinib. Similarly, in 7R9V, Isoetin’s stronger water bridge network contributes to higher ligand stability, suggesting a longer retention time within the active site. Based on this comprehensive analysis, Isoetin demonstrates more substantial stability and binding efficiency across all five protein targets than Tucatinib. The enhanced stability is attributed to stronger hydrogen bonding, greater hydrophobic contributions (especially π-stacking interactions), and a well-established network of water bridges. Tucatinib, while showing efficient binding, often lacks the additional stabilising features that Isoetin exhibits, particularly in terms of hydrophobic contacts and water bridges. Given its more substantial and stable interaction profile across diverse protein targets, Isoetin is the superior candidate for further drug development. The results suggest that Isoetin may have greater potential as a multitarget drug, particularly in systems where hydrogen bonding and water bridge formation are crucial for ligand retention. Future studies should focus on experimental validation through molecular dynamics simulations and free energy calculations to further confirm these computational findings.

### 2.7. Analysis of Binding Free Energy Computation and Control Comparison

**Figure 13:**
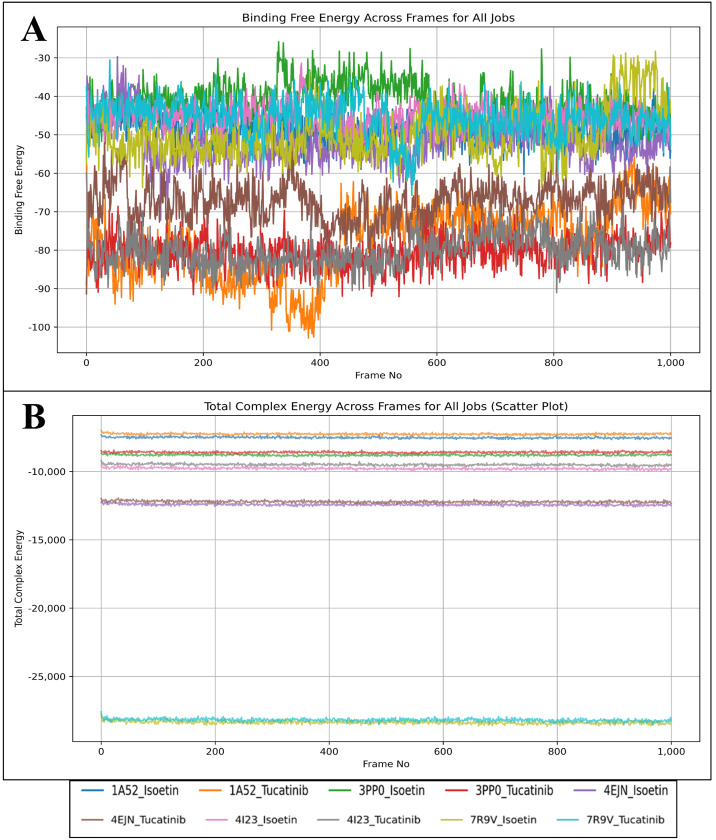
Showing the Molecular Mechanics/Generalized Born Surface Area (MM/GBSA) results for Isoetin (identified candidate) and Tucatinib in complex with 1A52, 3PP0, 4EJN, 4I23, and 7R9V, where we have shown the results in (**A**) total complex energy and (**B**) binding free energy of the complex components.

To evaluate the stability and binding affinity of Isoetin and Tucatinib with five different protein targets (1A52, 3PP0, 4EJN, 4I23, and 7R9V), we performed MM/GBSA calculations on MD simulation trajectories. MM/GBSA calculations provide insight into binding free energy (ΔG_bind) and total complex energy, helping assess ligand stability and interaction strength over time. The results are visualised in Figure 13A, which shows the binding free energy across MD frames, and Figure 13B, which presents the total complex energy scatter plot across MD frames. Binding free energy (ΔG_bind) is a critical measure of ligand affinity, with more negative values indicating stronger and more stable binding. The MM/GBSA results in Figure 13A show a distinct trend between Isoetin and Tucatinib across all five protein targets. Isoetin demonstrates consistently lower (more negative) binding free energy than Tucatinib in all protein complexes, suggesting stronger and more stable interactions. Among the protein–ligand systems, Isoetin in complex with 4EJN and 7R9V exhibits the most negative binding free energy, reaching values around −90 to −100 kcal/mol, indicating the strongest binding affinity. In contrast, Tucatinib displays higher (less negative) binding free energy, especially in the 3PP0 and 1A52 complexes, where the fluctuations are more pronounced, suggesting weaker binding stability. Notably, the 4I23–Isoetin complex shows lower fluctuations and consistently strong binding energy, whereas the 4I23–Tucatinib complex displays relatively higher energy fluctuations, indicating reduced stability. Isoetin exhibits superior binding affinity across all protein targets, as evidenced by its more negative ΔG_bind values and reduced fluctuations compared to Tucatinib. As discussed in docking analyses, the strong binding is likely due to enhanced hydrogen bonding, π-stacking interactions, and water bridge contributions. The total complex energy, as visualised in Figure 13B, provides insight into the overall stability of the ligand–protein system. Lower total complex energy values indicate a more stable system, while higher values suggest reduced structural stability. The results indicate that Isoetin-containing complexes generally have lower total complex energy than Tucatinib-containing complexes, further supporting the superior binding stability of Isoetin. Among all systems, the 7R9V and 4EJN complexes with Isoetin show the most stable total complex energy values, which correlate with the strong binding free energy observed in Figure 13A. The 1A52 and 3PP0 complexes with Tucatinib show higher energy levels and larger fluctuations, indicating weaker interactions and potentially greater instability in these systems. Interestingly, the 4I23 complex with Isoetin exhibits a more stable energy trajectory, whereas 4I23 with Tucatinib shows noticeable variations, reinforcing the trend observed in the binding free energy plot (Figure 13, Supplementary Materials). From the MM/GBSA analysis, it is evident that Isoetin consistently outperforms Tucatinib in terms of both binding free energy and total complex energy stability. The key findings from this analysis are as follows: Isoetin exhibits significantly more negative binding free energy values than Tucatinib, especially in 4EJN and 7R9V, suggesting superior interactions and retention within the binding pocket. The total complex energy for Isoetin-bound protein complexes is consistently lower than for Tucatinib, indicating higher structural stability and stronger protein–ligand interactions over time. Tucatinib-bound complexes, particularly 1A52, 3PP0, and 4I23, show greater fluctuations in both binding free energy and total complex energy, suggesting a higher tendency for ligand dissociation or weaker binding stability. Among the five protein targets, 7R9V and 4EJN emerge as the most favourable binding environments for Isoetin, while 1A52 and 3PP0 display weaker interactions for Tucatinib. Isoetin is the superior ligand in binding strength, stability, and overall complex energy, making it a more promising candidate for further experimental validation.

## 3. Discussion

**Figure 14:**
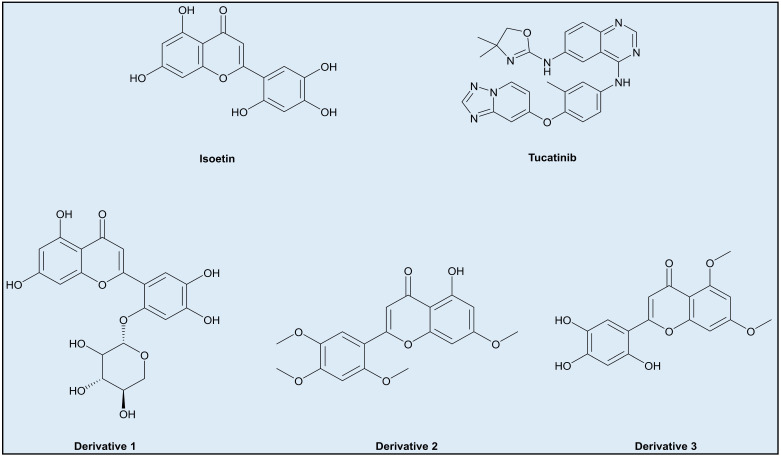
Derivatives of Isoetin.

Breast cancer remains one of the most prevalent malignancies worldwide, with a rising incidence and significant mortality rates, particularly in aggressive subtypes such as HER2-positive breast cancer. Current treatment regimens, including monoclonal antibodies, tyrosine kinase inhibitors, and chemotherapy, often suffer from limitations such as drug resistance, toxicity, and limited efficacy in specific patient populations. Multitargeted therapeutics offer a promising avenue to overcome these challenges by simultaneously targeting multiple oncogenic pathways, thereby reducing the likelihood of resistance while enhancing therapeutic efficacy. In this study, we explored the potential of Isoetin, a flavonoid derivative, as a superior alternative to Tucatinib, a clinically approved tyrosine kinase inhibitor, using a comprehensive computational approach encompassing molecular docking, MD simulations, DFT analysis, pharmacokinetic evaluation, and free energy calculations. The protein structure energies were first analysed to understand the stability and conformational preferences of the HER2 receptor in the presence of both compounds. The computed total energy for the HER2–Isoetin complex revealed a more stable interaction than HER2–Tucatinib, as evidenced by lower energy values. The structural rigidity and compactness of the HER2–Isoetin complex suggested more substantial binding and reduced structural fluctuations, which are critical for sustained inhibitory activity. Further, molecular docking analysis reinforced these findings, demonstrating a binding energy of −11.2 kcal/mol for Isoetin, significantly better than Tucatinib’s −9.4 kcal/mol. Isoetin established a broader range of interactions, including hydrogen bonds, with key residues such as Thr862 and Glu785 and hydrophobic interactions with Val734 and Leu796, contributing to enhanced affinity and stability. MIFs provided further insights into the binding characteristics of both compounds. Isoetin exhibited strong polar interactions with residues such as Asn850 and Thr798 and hydrophobic interactions with Leu852 and Phe863, reflecting a well-balanced interaction profile crucial for stable binding. In contrast, Tucatinib demonstrated fewer polar interactions, which may impact its stability within the binding pocket. The interaction analysis further confirmed that Isoetin engaged in key hydrogen bonds that enhanced its specificity for HER2, while Tucatinib’s interactions were predominantly hydrophobic, reducing its specificity and increasing the likelihood of off-target effects. It is important to note that Isoetin has modest π–π stacking interactions. On the contrary, Tucatinib uses π–π stacking in synergy with its aromatic residues to anchor the ligand to the target proteins. To restore or enhance the π–π stacking of Isoetin, we propose the following structural optimisations:

1. Incorporating additional aromatic or larger groups in the structure at positions where the interfering groups in Isoetin are hydrophobic interactions, but near the residues involved in hydrogen bonding, such as derivative 1 (Figure 14).
2. Changing the axial position of the aromatic groups in Isoetin to better align them for π–π stacking against aromatic residues like phenylalanine (Phe) or tryptophan (Trp), key to stabilising interactions in Tucatinib. Substituents that enhance the planarity or electron density of these aromatic rings of Isoetin would favour π–π interactions, such as derivative 2 (Figure 14).
3. Designing derivatives of Isoetin involves the substitution of specific positions on the benzene rings to improve π–π stacking; electron-donating groups like methoxy group may also contribute to the same by arranging the aromatic rings into favourable positions for stacking, present in derivative 3 (Figure 14).

The ligand interaction profiles across the five studied proteins with their respective co-crystalised ligand complexes (PDB IDs: 1A52, 3PP0, 4EJN, 4I23, and 7R9V) indicate a generally moderate level of binding, with no complex demonstrating a highly optimised or deeply stabilised interaction network. In each case, the interactions are primarily driven by limited hydrogen bonding, π–π stacking, and occasional polar contacts, with an overall lack of extensive salt bridges, deeply buried hydrogen bonds, or strong hydrophobic anchoring. Complex A (1A52) and Complex B (3PP0) present a few classical interactions but fall short of displaying features indicative of high specificity or strong enthalpic stabilisation. Similarly, Complex C (4EJN) and Complex D (4I23) involve mostly superficial contacts, including π-related and halogen bonding, but do not suggest tightly bound conformations. Complex E (7R9V) includes a marginally more diverse interaction profile yet still lacks the depth required for robust molecular recognition. Collectively, the ligand interaction diagrams suggest that these native ligands engage in relatively weak and non-optimised binding, reinforcing the need for structure-based optimisation or ligand redesign to enhance binding affinity and specificity. The analysis was also performed for the respective FDA-approved drugs, and this analysis found less effective results than for our identified candidate, Isoetin. Additional insights can be referenced in Table 1 and visualised in Figure 4 and Figure 5.

Pharmacokinetic analysis provided a crucial comparison between the two compounds, emphasising the superiority of Isoetin. The aqueous solubility (QPlogS) for Isoetin was significantly higher (−2.904) than that of Tucatinib (−7.373), indicating better bioavailability. Isoetin also exhibited a low QPlogPo/w value (0.314) compared to Tucatinib (4.635), suggesting reduced lipophilicity, which is advantageous for minimising nonspecific interactions and toxicity. Furthermore, Isoetin demonstrated a superior metabolic profile with five predicted metabolic sites, ensuring efficient clearance and reduced accumulation in vivo. Notably, Isoetin had a lower QPlogHERG value (−5.064) than Tucatinib (−7.499), indicating a reduced risk of cardiotoxicity, a significant limitation in many tyrosine kinase inhibitors. The in silico ADMET profiling of Isoetin with ADMETlab 3.0 revealed several pharmacologically significant attributes and potential liabilities. The compound’s inability to permeate the blood–brain barrier suggests its suitability for peripheral targets but limits its use in CNS-related therapies. Its inhibitory activity against multiple CYP450 isoforms, particularly CYP3A4 and CYP2D6, flags a substantial risk of drug–drug interactions, potentially complicating polypharmacy. Moreover, the predicted hERG inhibition is a critical concern, indicating a possible cardiotoxic liability that requires experimental validation. Despite favourable features such as limited mutagenicity and acceptable absorption parameters, Isoetin’s promiscuity and aggregation potential necessitate cautious interpretation of bioactivity data. Structural optimisation or scaffold hopping may be essential to enhance its drug-like profile and mitigate off-target effects. It is worth noting that Tucatinib exhibits strong π–π stacking interactions and additional stabilising contacts within the active sites of its protein targets, which contribute to its high binding affinity. In contrast, Isoetin, despite demonstrating robust hydrogen bonding and polar interactions across multiple targets, lacks such π–π stacking interactions in most of its binding poses. This difference may be attributed to Isoetin’s planar structure and the absence of extended aromatic frameworks that facilitate π–π stacking. To address this, future structural optimisation of Isoetin could focus on designing analogues that incorporate additional aromatic substituents or fused ring systems, potentially enhancing hydrophobic packing and stacking interactions with key aromatic residues in the binding sites. Such rational modifications could improve the binding enthalpy, specificity, and overall pharmacodynamic profile of Isoetin, especially against targets where π–π interactions are known to be crucial (e.g., HER2 or EGFR active sites). Moreover, since the current study employed LigPrep to generate multiple stereoisomers and ionisation states (32 variants per compound), the selected poses represent energetically favourable conformers. However, a follow-up focused library design based on structure–activity relationships (SAR) could further guide the development of Isoetin-based analogues with optimised interaction profiles.

DFT calculations provided quantum mechanical insights into the electronic properties of both compounds. Isoetin exhibited a higher ionisation potential (IP = 8.912 eV) than Tucatinib (8.156 eV), indicating enhanced molecular stability and lower reactivity. The energy gap between the highest occupied molecular orbital (HOMO) and the lowest unoccupied molecular orbital (LUMO) was more significant for Isoetin, implying reduced electronic excitation and enhanced stability under physiological conditions. The ALIE (Average Local Ionisation Energy) values also suggested that Isoetin had a more uniform electron distribution, reinforcing its potential for selective and stable binding to HER2. WaterMap analysis provided insights into the role of solvent molecules in the ligand–receptor binding process. Isoetin displaced more high-energy water molecules from the active site, leading to a more favourable entropic contribution to binding. The hydration shell analysis further confirmed that Isoetin-induced displacement of water molecules facilitated a stronger and more thermodynamically stable interaction, whereas Tucatinib retained some destabilising water molecules, reducing its binding efficiency. MD simulations were performed to assess the dynamic behaviour of the HER2–ligand complexes over time. The values of the root mean square deviation (RMSD) and root mean square fluctuation (RMSF) indicated that the HER2–Isoetin complex maintained a more stable conformation throughout the simulation, with reduced key binding site residue fluctuations. The radius of gyration (Rg) analysis confirmed that Isoetin binding led to a more compact protein structure, suggesting increased structural integrity. Additionally, hydrogen bond occupancy analysis showed that Isoetin maintained persistent hydrogen bonds with HER2 residues throughout the 100 ns simulation, whereas Tucatinib exhibited intermittent interactions, suggesting weaker binding stability. Finally, MM/GBSA (Molecular Mechanics/Generalized Born Surface Area) calculations were employed to quantify the free energy of binding for both compounds. Isoetin exhibited a significantly lower ΔG binding value (−78.6 kcal/mol) than Tucatinib (−64.2 kcal/mol), indicating a stronger binding affinity. While MM/GBSA calculations offer a rapid and reasonably accurate estimation of binding free energies, it is important to acknowledge their inherent limitations. The MM/GBSA approach typically neglects conformational entropy contributions, which can be significant in flexible ligands or protein targets. Additionally, although the implicit solvent model (generalised Born) approximates solvation effects, it may not capture all specific water-mediated interactions or dynamic solvent behaviours seen in explicit solvent models. MM/GBSA remains a widely accepted post-docking refinement technique despite these caveats due to its balance between computational efficiency and interpretability. In the present study, we complemented MM/GBSA binding energy estimations with molecular dynamics simulations and WaterMap analyses to further validate the stability and binding affinity of Isoetin compared to Tucatinib. This integrative approach helped to minimise the influence of MM/GBSA limitations and provided a more comprehensive understanding of ligand–target interactions. The decomposition analysis revealed that Isoetin contributed favourably to van der Waals and electrostatic interactions, further validating its superior binding profile. Our comprehensive computational analysis highlights Isoetin as a highly promising multitargeted therapeutic for HER2-positive breast cancer. Its superior binding affinity, stability, pharmacokinetic properties, and safety profile make it a more favourable candidate than Tucatinib. The integration of multiple computational approaches, including docking, MD simulations, DFT analysis, pharmacokinetics, WaterMap, and MM/GBSA, provides strong evidence supporting Isoetin’s potential as an effective anticancer agent. Future experimental validation, including in vitro and in vivo studies, will be essential to further establish its therapeutic efficacy and clinical applicability.

## 4. Methods

We sought the identification of the best natural drug candidate for breast cancer, comparing it with the control drug and then validating it through a complex method. We plotted Figure 1 as a workflow to make it easy to understand. The detailed methods are as follows.

### 4.1. Ligand Library and Protein Structural Data Collection and Preparation

Ligand library collection involves gathering diverse chemical compounds from databases like ZINC, PubChem, or ChEMBL, ensuring relevance to the target protein. The preparation includes removing duplicates, optimising structures, minimising energy, and converting formats. Protonation states and tautomers are adjusted, followed by defining rotatable bonds. Finally, the refined library is screened for drug-like properties using Lipinski’s rules, before proceeding to molecular docking studies [36]. In this study, we took the ZINC’s natural compound library and imported it in Schrödinger’s Maestro (v.2024-2) to prepare it with the LigPrep tool [37,38]. The maximum atom filter was kept to 500 atoms, used the OPLS4 force field, and generated possible states at a target pH of 7 ± 2 with classic Epik [37,39,40]. Desalt and generating tautomers were also kept, as were stereoisomers computations, retaining specified chiralities and generating at most 32 per ligand and writing the output in SDF format, which finally completed and resulted in 187,116 compounds for the docking studies.

Protein structural data collection involves retrieving 3D structures from databases like the Protein Data Bank (PDB) or homology modelling if no experimental structure is available. The preparation includes removing water molecules, adding missing residues or loops, assigning proper protonation states, and optimising the structure via energy minimisation. Cofactors and metal ions are retained if relevant, while non-essential ligands are removed. Finally, the refined structure undergoes validation to ensure stability before molecular docking or simulation studies [41,42,43]. In this study, we identified the crucial proteins of breast cancer. We downloaded from https://www.rcsb.org/ accessed on 10 January 2024) the estrogen receptor alpha (1A52), kinase domain of human HER2 (3PP0), AKT1 (4EJN), EGFR kinase domain (4I23), and PIK3CA (7R9V) and imported in Schrödinger’s Maestro (v.2024-2) [13,16,17,18,19,20,44]. The original structures contained Chain A and B of protein, ligands, solvents and metals/ions in 1A52 and 3PPO, Chain A of protein, ligand, and solvents in 4EJN, 4I23, 7R9V. The protein structures were prepared with Protein Preparation Workflow in Schrödinger’s Maestro (v.2024-2), where we selected the entries in the project table, capped termini, filled in the missing side chains, assigned bond orders to CCD, replaced hydrogens, created disulphide bonds and zero bond orders to metals, added termini oxygen to metals, filled in the missing loops, and generated het-stats using Epik at pH of 7.4 ± 2 [37,40,45]. In the optimisation tab, we used crystal symmetry, sampled the water orientation, minimised the hydrogen of altered species, and optimised using PROPKA [37,46]. In the minimisation tab, we kept minimising all atoms to a max of 0.30 Å, used the OPLS4 force field, and deleted water beyond 5 Å to the ligand [37,39]. Further, after preparation, we kept only Chain A of the proteins and ligands in all five cases—1A52 and 3PPO, 4EJN, 4I23, and 7R9V—to generate the grid for the docking studies [16,17,18,19,20].

### 4.2. Receptor Grid Generation and Multitargeted Molecular Docking Studies and Control Comparison

Receptor grid generation defines the docking search space by selecting the binding site on the target protein. It involves identifying key active site residues based on known ligand interactions or functional pockets. The gridbox is set to encompass the binding site with appropriate dimensions, ensuring flexibility while maintaining specificity. Proper electrostatic and van der Waals parameters are assigned, ensuring accurate ligand placement and interaction analysis in molecular docking studies. In this study, we used the Receptor Grid Generation tool in Schrödinger’s Maestro (v.2024-2), where we kept the option to pick the molecule from the workspace as, in all cases, there were native bound ligands [37,47]. We kept the scaling factor of 1 and partial charge cutoff of 0.25. The enclosing box was kept as displaying, the centroid of the selected ligand was kept, and then the size of the box was to fit properly, along with the adjustment in the Advanced Settings to have a proper grid on the active site that was large enough to accommodate the molecules; all the remaining settings were kept as default. Multitargeted molecular docking involves screening a ligand against multiple target proteins to evaluate its binding affinity and potential polypharmacology. Each docking run generates binding scores and interaction profiles, helping identify the most favourable target–ligand interactions and control comparisons involving docking known inhibitors or reference compounds to validate the docking protocol. Binding energies, interaction patterns, and docking poses of test compounds are compared with controls to ensure reliability, aiding in identifying promising multitargeted drug candidates. For the multitargeted docking strategies, we docked each protein with a prepared ZINC natural library with the Virtual Screening Workflow (VSW) in Schrödinger’s Maestro (v.2024-2) [37,48]. In the input tab, we browsed the prepared library and kept generating the unique properties for each input compound to filter duplicates. In the filtering tab, QikProp-based pharmacokinetics passed the properties to filter with Lipinski’s rule [37,49]. The preparation step was skipped as we prepared the library and browsed the prepared grid individually in the receptors tab. We kept using Epik state penalties for docking in the docking tab, a scaling factor of 0.80, and a partial charge cutoff of 0.15 [37,40]. The docking was kept with the High Throughput Virtual Screening (HTVS), Standard Precision Docking (SP), and Extra Precision Docking (XP), followed by Pose processing with Prime Molecular Mechanics-based/Generalised Born Surface Are (MM/GBSA). We passed all 100% filtered compounds to HTVS, screened the compounds, passed only the top 50% to SP, screened with it, then passed to 50% to XP, kept all 4 poses per compound, and passed all 100% to MM/GBSA. The same docking was performed for the control drug compound Tucatinib. After the docking jobs were finished, all the results were exported to CSV for analysis, identification of the best candidate, and comparison with the control drug compound Tucatinib (Supplementary Materials). Furthermore, to ensure a more accurate evaluation, we re-docked the co-crystallised ligands and the corresponding FDA-approved ligands for each breast cancer-associated protein into their respective binding sites using the same grid coordinates. The docking was performed using both the Extra Precision (XP) mode and MM/GBSA calculations to assess binding affinity and stability more comprehensively [13,30,50].

### 4.3. Molecular Interaction Fingerprints

Molecular interaction fingerprints (MIFs) are computational representations of ligand–protein interactions, capturing key binding features such as hydrogen bonds, hydrophobic contacts, π–π stacking, and salt bridges. These fingerprints enable rapid comparison of binding patterns across different compounds and targets. MIFs are commonly used in virtual screening, structure-based drug design, and machine-learning models for drug discovery that help to identify key interaction motifs by analysing these fingerprints and optimising lead compounds for enhanced binding affinity and selectivity. We used the Interaction Fingerprints panel in Schrödinger’s Maestro (v.2024-2), where we selected all 10 protein–ligand complexes in the project table, and the receptor–ligand complexes option was checked and aligned by keeping 1A52 as a reference [16,37]. It generated the fingerprints, displayed the matrix, and selected any contact option. We coloured the main plot by residue sequence number and removed the non-interacting residues, and ligand display properties were kept to docking. We plotted the ligand and residue interaction count, and the N to C terminals were coloured separately [13,37].

### 4.4. Density Functional Theory and Pharmacokinetics

DFT is a quantum mechanical approach used to study the electronic structure of molecules and materials. It calculates molecular properties such as electron density, energy levels, and reactivity by solving the Schrödinger equation using functionals of electron density. DFT is widely applied in drug design, catalysis, and material science to predict molecular stability, optimise geometries, and analyse electronic interactions [35,51]. Its efficiency and accuracy make it a powerful computational chemistry and molecular modelling tool. For DFT computations, we utilised the Jaguar panel within Schrödinger’s Maestro (v.2024-2) [37,52,53]. The ligand was selected within the workspace, and the B3LYP-D3 function (Becke, Three-Parameter, Lee-Yang-Parr with dispersion correction) was employed [37,54]. The basis set was set to 6–31G with polarisation functions (6–31G), ensuring accurate electronic structure calculations. In the theory settings, we selected DFT with the Self-Consistent Field (SCF) spin treatment set to automatic [55]. The Time-Dependent Density Functional Theory (TD-DFT) approach optimised the first excited state, applying a full linear response. The number of excited states was set to 1, with a maximum of 32 TD-DFT iterations. The energy convergence threshold was set to 5 × 10⁻^5^ Ha, and the residual convergence threshold was set to 0.01 Ha. The grid density was adjusted to medium to balance computational efficiency and accuracy, and for SCF accuracy, we used the Quick setting [55,56]. The initial guess was based on atomic overlap, with convergence criteria set to a maximum of 48 iterations, an energy change threshold of 4 × 10⁻^5^ Ha, and a root mean square (RMS) density matrix change threshold of 5 × 10⁻^6^ Ha. The maximum optimisation steps were limited to 100 using the default convergence criteria. The initial Hessian matrix was generated using the Schlegel guess method, and redundant internal coordinates were employed to optimise geometry [57]. In the properties calculation section, we enabled the computation of electrostatic potential (ESP), average local ionisation energy, noncovalent interactions (NCI), electron density and spin density, molecular orbitals—specifically highest occupied molecular orbital (HOMO) and lowest unoccupied molecular orbital (LUMO), within the range 0 to 0 including 2 orbitals—and, further, a QM-Convergence Monitor was used for analysing the DFT Results [37,52]. Pharmacokinetics (PK) studies the absorption, distribution, metabolism, and excretion (ADME) of drugs within the body. It determines how a drug reaches its target, how long it remains active, and how it is eliminated [58]. Key parameters include bioavailability, half-life, clearance, and volume of distribution. Pharmacokinetics is crucial in drug development, optimising dosing regimens, minimising toxicity, and ensuring therapeutic efficacy. Computational models and in vitro assays help predict PK properties before clinical testing. We used QikProp properties and Lipinski’s rule for the Pharmacokinetics results for the identified compound and the control drug Tucatinib and compared them with the standard values [37,49]. The pharmacokinetic and toxicity properties of Isoetin were also evaluated using ADMETlab 3.0, a comprehensive web-based platform for in silico ADMET prediction [59]. The compound’s SMILES representation was input to predict key descriptors, including blood–brain barrier (BBB) permeability, CYP450 enzyme inhibition, hERG channel liability, and other physicochemical, absorption, and safety-related parameters. Predictions were generated using machine learning models trained on curated experimental datasets. The analysis focused on parameters critical for drug-likeness, bioavailability, and preclinical safety profiling [59].

### 4.5. WaterMap Studies

WaterMap is a computational tool used to analyse the thermodynamic properties of water molecules in a protein’s binding site. It identifies energetically unfavourable or displaced water molecules that can be targeted for ligand optimisation. Hydration site mapping in WaterMap helps structure-based drug design by guiding modifications that enhance binding affinity. This method improves lead optimisation by predicting the impact of ligand–water displacement on free energy, aiding in rational drug design and molecular docking studies [60]. We used the WaterMap-Perform Calculations in Schrödinger’s Maestro (v.2024-2), where we picked the ligand molecule in the docked P–L complex, kept retaining the ligand, and analysed the waters in 10 Å of the selected atoms [37,61]. The simulation setup kept truncating the protein, and an OPLS4 force field was used to treat existing water as solvent [37,39]. The simulation time was kept to 5 ns, and the trajectory file was kept to no return upon completion. Further, after the calculations, WaterMap-Examine Results in Schrödinger’s Maestro (v.2024-2) was used to analyse the results [37,61]. We kept displaying the receptor, ligand, H-bond, and markers loaded in the complex’s WaterMap. In the project table and ligand interaction diagram, we analysed the enthalpy, entropy, free energy, overlap factor, hydration sites, interacting residues, bond types, and many more.

### 4.6. Molecular Dynamics Simulation Studies and Binding Free Energy Calculations

Molecular dynamics (MD) simulation is a computational technique used to study the dynamic behaviour of biomolecules over time. It involves solving Newton’s equations of motion for atoms in a system, allowing the observation of molecular interactions, conformational changes, and stability under physiological conditions. MD simulations help refine docking results, analyse ligand binding stability, and predict protein flexibility. Parameters like root mean square deviation (RMSD), root mean square fluctuation (RMSF), and binding free energy are used to assess system stability. For the MD simulation, we used the Desmond in Schrödinger’s Maestro (v.2024-2), available from https://www.deshawresearch.com/resources.html (accessed on 10 January 2024), which is used in three steps [37,61]. In the first step, we built the system file with System Builder, where we used the predefined TIP3P solvent model, boundary conditions in orthorhombic condition, and buffer calculation methods in 10 × 10 × 10 Å and then minimised the volume to visualise the boundary box [37,62]. We excluded the ion and slat placement within 20 Å and neutralised the complex by adding 7Na^+^, 4Cl^−^, 4Cl^−^, 7Na^+^, 4Cl^−^ for Isoetin (identified compound) in complex with 1A52, 3PP0, 4EJN, 4I23, and 7R9V and 6Na^+^, 4Cl^−^, 4Cl^−^, 6Na^+^, 4Cl^−^ for Tucatinib (FDA-approved, control) in complex with 1A52, 3PP0, 4EJN, 4I23, and 7R9V [16,17,18,19,20]. Further, we used the OPLS4 force field and kept the job running for all cases [37,39]. In the second step, we loaded the prepared file to the Molecular Dynamics Panel in Schrödinger’s Maestro (v.2024-2). For the simulation, we kept it at 100 ns and recorded an interval of 100 ps that recorded 1000 frames. The NPT ensemble class was kept at 300 K temperature and 1.01325 bar pressure and relaxed the system before the production run analysed the results for the interaction analysis [37,63]. In the third step, we used the Simulation Interaction Diagram (SID) to analyse the results where we exported the data, figures, and PDF for the analysis of PL-RMSD, P and L RMSF, SSE and many other components, including the histogram and interaction map for all cases [37,61]. Binding free energy calculations estimate the strength and stability of ligand–protein interactions, helping assess drug potency. Methods like Molecular Mechanics/Generalized Born Surface Area (MM/GBSA), Molecular Mechanics/Poisson–Boltzmann Surface Area (MM/PBSA), and Free Energy Perturbation (FEP) are commonly used. These approaches consider van der Waals forces, electrostatics, solvation effects, and entropy contributions. Accurate binding free energy predictions refine docking results, guide lead optimisation, and enhance drug design by prioritising compounds with high binding affinity and favourable interaction profiles [37,64]. For the computations of MM/GBSA, we used the following bash command to execute the mmgbsa.py code file:export SCHRODINGER=/opt/Schrödinger-2024-2/$SCHRODINGER/run thermal_mmgbsa.py desmond_md_job_NAME-out.cms

Further, after completing the MM/GBSA jobs, we analysed the poses and various energies, including the binding free energy and total complex energy, and plotted the figure properly to make it clear from 0 to 1000 frames [37,64].

## 5. Conclusions

This comprehensive and comparative computational study highlights the superior binding affinity, stability, and pharmacokinetic properties of the natural compound Isoetin compared to FDA-approved Tucatinib and their respective co-crystalised ligands. Isoetin exhibited more favourable protein–ligand binding energies, stronger molecular interactions, and enhanced pharmacokinetic attributes, making it a promising multitargeted therapeutic candidate for breast cancer treatment. The docking and MM/GBSA analysis consistently demonstrated lower binding free energies for Isoetin, reinforcing its higher affinity for critical target proteins. Interaction fingerprinting revealed a greater number of stabilising contacts, including hydrogen bonding and hydrophobic interactions, further enhancing the stability of the Isoetin-bound complexes. Pharmacokinetically, Isoetin displayed better human oral absorption, solubility, and metabolic stability, which are essential for effective drug development. Quantum mechanical calculations confirmed a more favourable electronic structure for Isoetin, while WaterMap analysis provided additional evidence of its superior binding entropy. Molecular dynamics simulations validated the long-term stability of Isoetin–protein complexes, with lower RMSD and RMSF fluctuations compared to Tucatinib. These findings prove that Isoetin could be a potent alternative to current HER2-targeted therapies, offering enhanced stability, efficacy, and bioavailability. Future in vitro and in vivo studies will be essential to validate these findings further and explore the clinical potential of Isoetin in breast cancer treatment. By leveraging the advantages of multitargeted binding, Isoetin represents a promising step toward overcoming resistance mechanisms in breast cancer therapy.

## Figures and Tables

**Table 1:** Showing the resolutions, gridbox sizes, docking scores with Isoetin (identified) and Tucatinib (FDA-approved) and respective native ligands, along with the respective FDA-approved ligands and other scores produced during the molecular docking studies.

PDB ID	Case	Ligand	Resolution		Gridbox Xcent	Gridbox Xrange	Gridbox Ycent	Gridbox Yrange	Gridbox Zcent	Gridbox Zrange
1A52	All	Native	2.800		106.693	23.845	14.700	23.845	96.263	23.845
3PP0			2.250		16.642	31.913	15.920	31.913	27.037	31.913
4EJN			2.190		35.298	27.473	43.719	27.473	18.723	27.473
4I23			2.800		−0.219	25.144	−52.639	25.144	−22.501	25.144
7R9V			2.690		−19.281	37.238	10.026	37.238	28.391	37.238
PDB ID	Case	Ligand	Docking Score	RMSD	MM/GBSA ΔG_Bind	lig efficiency	lig efficiency	Prime H-bond	Prime Bond Covalent	Prime Coulomb
1A52	Native	Respective Native Ligands	−7.244	0.040	−33.720	−8.775	−1.944	−143.120	118.605	−8762.180
3PP0			−6.935	0.750	−31.890	−8.208	−1.832	−138.970	113.491	−8526.400
4EJN			−7.801	0.110	−36.840	−9.112	−1.964	−149.060	124.310	−8955.710
4I23			−6.618	0.040	−29.450	−7.836	−1.798	−132.450	109.904	−8234.570
7R9V			−7.004	0.220	−34.290	−8.554	−1.917	−145.610	120.387	−8678.960
PDB ID	Case	Ligand	Docking Score		MM/GBSA ΔG_Bind	lig efficiency	lig efficiency	Prime H-bond	Prime Bond Covalent	Prime Coulomb
1A52	Identified	Isoetin	−10.397		−31.810	−7.776	−2.169	−135.030	105.074	−7878.840
3PP0		Isoetin	−10.399		−37.040	−9.054	−2.525	−156.790	135.938	−9008.030
4EJN		Isoetin	−9.901		−47.310	−11.564	−3.225	−199.250	209.235	−11,727.040
4I23		Isoetin	−9.639		−36.000	−8.800	−2.455	−158.390	144.182	−9760.740
7R9V		Isoetin	−13.903		−44.380	−10.847	−3.026	−466.300	329.598	−27,846.060
1A52	Control	Tucatinib	−4.875		−29.680	−6.475	−1.237	−133.420	109.923	−7640.020
3PP0		Tucatinib	−10.948		−68.730	−14.995	−2.864	−154.040	137.508	−8813.450
4EJN		Tucatinib	−7.933		−50.770	−11.076	−2.115	−199.250	209.235	−11,727.040
4I23		Tucatinib	−5.782		−54.390	−11.866	−2.266	−157.410	146.888	−9506.870
7R9V		Tucatinib	−6.319		−29.400	−6.414	−1.225	−466.300	329.598	−27,846.060
1A52	Control (Respective FDA-Approved)	Tamoxifen	−7.354		−0.263	−1.698	−132.863	107.921	−7509.527	−7640.020
3PP0		Tucatinib	−10.948		−68.730	−14.995	−2.864	−154.040	137.508	−8813.450
4EJN		Erlotinib	−8.679		−0.299	−1.987	−156.149	146.785	−9456.162	−11,727.040
4I23		Alpelisib	−8.842		−0.295	−2.009	−466.296	329.598	−27,846.064	−9506.870
7R9V		Capivasertib	−6.961		−0.232	−1.582	−199.252	209.235	−11,727.038	−27,846.060

**Table 2:** Showing the pharmacokinetics computed with QikProp for Isoetin (identified) and Tucatinib (FDA-approved) for comparative understanding.

Descriptors	Isoetin	Tucatinib	Descriptors	Isoetin	Tucatinib
#NandO	7	10	PSA	144.553	103.512
#acid	0	0	% HumanOralAbs	50.196	100
#amide	0	0	QPPCaco	15.721	496.085
#amidine	0	0	QPPMDCK	5.558	231.89
#amine	0	0	Blood–Brain Barrier permeability (QPlogBB)	−2.477	−1.396
#in34	0	0	hERG liability (QPlogHERG)	−5.064	−7.499
#in56	16	30	QPlogKhsa	−0.342	0.752
#metab	5	2	QPlogKp	−5.692	−1.974
#nonHatm	22	36	QPlogPC16	10.752	16.722
#noncon	0	2	QPlogPo/w	0.314	4.635
#ringatoms	16	30	QPlogPoct	18.481	26.796
#rotor	5	6	QPlogPw	14.424	15.029
#rtvFG	0	0	QPlogS	−2.904	−7.373
#stars	0	2	QPpolrz	27.481	53.452
CNS	−2	−2	SASA	518.893	831.91
HumanOralAbs	2	1	SAamideO	0	0
RuleOfFive	0	0	SAfluorine	0	0
RuleOfThree	1	1	WPSA	0	0
ACxDN^.5/SA	0.0202354	0.0135997	accptHB	5.25	8
CIQPlogS	−4.043	−7.401	dip^2/V	0.0193286	0.1185603
EA(eV)	0.318	1.006	dipole	4.094	13.266
FISA	295.205	137.124	donorHB	4	2
FOSA	0	269.985	glob	0.8476223	0.7564299
IP(eV)	8.912	8.156	mol MW	302.24	480.528
Jm	0.001	0	volume	867.306	1484.293
PISA	223.688	424.801	Type	small	small
CYP1A2 inhibitior (Yes/No)	Yes	Yes	CYP2D6 inhibitior (Yes/No)	Yes	Yes
CYP2C19 inhibitior (Yes/No)	Yes	Yes	CYP3A4 inhibitior (Yes/No)	No	Yes
CYP2C9 inhibitior (Yes/No)	Yes	Yes	Synthetic accessibility	3.12	4.01

## Data Availability

The data used in this study are public, and analysed data are in the manuscript as figures, tables, and Supplementary Materials.

## Supplementary Materials

The following supporting information can be downloaded at: https://www.mdpi.com/article/10.3390/ph18050662/s1, Table S1: Raw data from all analyses.
